# The Diacylglycerol Analogs OAG and DOG Differentially Affect Primary Events of Pheromone Transduction in the Hawkmoth *Manduca sexta* in a Zeitgebertime-Dependent Manner Apparently Targeting TRP Channels

**DOI:** 10.3389/fncel.2018.00218

**Published:** 2018-07-24

**Authors:** Petra Gawalek, Monika Stengl

**Affiliations:** Animal Physiology, FB 10, Biology, University of Kassel, Kassel, Germany

**Keywords:** transient receptor potential ion channels, insect olfaction, olfactory transduction, diacylglycerol, tip recordings, olfactory sensilla

## Abstract

For the hawkmoth *Manduca sexta* accumulating evidence suggests that pheromone transduction acts via a metabotropic signal transduction cascade, with G-protein-dependent phospholipase C (PLC) activations generating diacylglycerol (DAG) and inositol trisphosphate as the primary events in hawkmoth pheromone transduction. In contrast, ionotropic olfactory receptor (OR) coreceptor (Orco)-dependent mechanisms do not appear to be involved. In hawkmoths pheromones activated a specific sequence of PLC-dependent ion channels of unknown identity. In several sensory systems transient receptor potential (TRP) ion channels were found downstream of PLC as primary transduction channels. Also in the mammalian vomeronasal organ, DAG-dependent TRP channels are employed. Therefore, we hypothesized that TRPs may be downstream targets for DAG also in the hawkmoth pheromone signal transduction pathway. To test this, we employed two DAG analogs, OAG and DOG for *in vivo* single-sensillum tip-recordings of pheromone-sensitive sensilla. Since olfactory receptor neurons (ORNs) expressed circadian changes in sensitivity throughout the day, we recorded at two different Zeitgebertimes (ZTs), the hawkmoths activity phase at ZT 1 and its resting phase at ZT 9. We found that the DAG analogs targeted at least two different TRP-like channels that underlie the primary events of hawkmoth pheromone transduction daytime-dependently. At both ZTs OAG sped up and increased the Orco-independent phasic action potential response without affecting the Orco-dependent late, long-lasting pheromone response. Thus, OAG most likely opened a transient Ca^2+^ permeable TRP channel that was available at both ZTs and that opened pheromone-dependently before Orco. In contrast, DOG slowed down and decreased the sensillum potential, the phasic-, and the late, long-lasting pheromone response. Therefore, DOG appeared to activate a protein kinase C (PKC) that closed TRP-like Ca^2+^ permeable channels and opened Ca^2+^ impermeable cation channels, which have been previously described and are most abundant at ZT 9. These data support our hypothesis that hawkmoth pheromone transduction is mediated by metabotropic PLC-dependent mechanisms that activate TRP-like channels as the primary event of pheromone transduction. In addition, our data indicate that at different times of the day different second messenger-dependent ion channels are available for pheromone transduction cascades.

## Introduction

The circadian release of pheromones synchronizes hawkmoths physiology and behavior (Riffell et al., 2008; Schendzielorz et al., 2015). Females of the nocturnal hawkmoth *Manduca sexta* attract their mates at night. They release their species-specific sex-pheromone blend in a strictly circadian rhythm (Sasaki and Riddiford, 1984; Tumlinson et al., 1989). Also the male's sensitivity to detect these pheromones expresses a circadian rhythm, governed by fluctuating hormone levels (review: Stengl, 2010). With long trichoid sensilla on their antennae male hawkmoths detect the sex-pheromone blend over about 8 log units of concentrations during their activity phase at night, while they are considerably less sensitive during the day when moths are at rest (Dolzer et al., 2003; Flecke and Stengl, 2009; Flecke et al., 2010). Each trichoid sensillum is innervated by two olfactory receptor neurons (ORNs) that extend their dendritic cilia into the long hair shaft (Sanes and Hildebrand, 1976; Keil and Steinbrecht, 1984; Keil, 1989). One of the ORNs responds to bombykal, the main sex pheromone component, while the other is sensitive to other components of the pheromone blend (Kaissling et al., 1989). The pheromones are detected via specified pheromone receptors on the cilia of the ORNs that were cloned before, but their functions were still not understood (Große-Wilde et al., 2010; Wicher et al., 2017; reviews: Nakagawa and Vosshall, 2009; Stengl, 2010, 2017). The goal of this study is to delineate the signaling pathway by which pheromones activate hawkmoth ORNs. The large hawkmoths are an established model system for olfaction and are better suited to physiological studies as compared to the tiny fruitflies that instead are excellent genetic model organisms.

### Insect odor transduction is still under debate

*Drosophila melanogaster* ORs are inverse 7-transmembrane receptors with an intracellular N-terminus (Benton et al., 2006; Lundin et al., 2007) that heteromerize with the conserved olfactory receptor coreceptor (Orco) (Vosshall and Hansson, 2011). Orco is necessary for the localization and maintenance of ORs in the ciliary membranes of *Drosophila* ORNs (Larsson et al., 2004; Benton et al., 2006). Next to this “chaperon function” of Orco it forms a spontaneously opening ion channel controlling the spontaneous activity of ORNs in different species (Larsson et al., 2004; Benton et al., 2007; Sato et al., 2008; Wicher et al., 2008; Deng et al., 2011; Jones et al., 2011; Sargsyan et al., 2011; Nolte et al., 2013). Despite of extensive molecular genetic studies, it is still not agreed upon whether or how Orco is directly involved in the primary events of insect odor transduction. Thus, it is still not resolved which ion channels underlie the receptor potential that triggers pheromone-dependently action potentials (APs) in insect ORNs (Jones et al., 2011; Sargsyan et al., 2011; Nolte et al., 2013, 2016; reviews: Nakagawa and Vosshall, 2009; Stengl, 2010, 2017).

### Hawkmoth pheromone transduction involves G-protein-dependent activation of phospholipase Cβ

Patch clamp studies of *M. sexta* ORNs in primary cell culture combined with pharmacology characterized several bombykal- and second messenger-dependent ion channels in ORNs with properties of transient receptor potential (TRP)-like ion channels (Minke et al., 1975; Montell et al., 1985; Stengl and Hildebrand, 1990; Stengl et al., 1992; Stengl, 2010). These studies showed that pheromone activates phospholipase C (PLC) in a G-protein dependent manner. This generated diacylglycerol (DAG) and inositol-trisphosphate (IP_3_) as a function of pheromone concentration as measured in different insect antennae (Boekhoff et al., 1990, 1993; Breer et al., 1990). Infusion of GTPγS or IP_3_ opened the same sequence of TRP/TRPL-like ion channels as does application of pheromone (Stengl, 1993, 1994; Dolzer et al., 2001; review: Stengl, 2010). In the presence of adapting concentrations of pheromone DAG and elevated Ca^2+^-levels activated protein kinase C (PKC) that closed the previously opened pheromone-dependent channels (Stengl, 1993, 1994; Dolzer et al., 2001; review: Stengl, 2010). Thus, depending on pheromone concentration, different second messenger cascades become active. It has been established previously that DAG directly opens odor-dependent Ca^2+^-permeable TRPC2- channels in vertebrate ORNs (Lucas et al., 2003). Also TRP channels are closed via PKC-dependent phosphorylation (Huang, 1989; Liu and Heckman, 1998; Venkatachalam et al., 2003). Therefore, DAG has at least two different antagonistic functions. It can directly open TRP channels causing membrane potential depolarizations and it can directly activate PKC which is involved in negative feedback regulation, closing TRP channels and curtailing membrane potential depolarizations.

### Directly- or indirectly DAG-dependent ion channels belong to the transient receptor potential (TRP) superfamily of ion channels

The superfamily of TRP ion channels comprises 7 subfamilies of evolutionary highly conserved cation channels first detected in fly vision (Minke et al., 1975; Montell et al., 1985; Hofmann et al., 1999; reviews: Ramsey et al., 2006; Fowler and Montell, 2013). The subfamily of classical or canonical TRPs (TRPCs) are the closest mammalian homologs of *Drosophila melanogaster* TRP/TRP-like Ca^2+^ permeable cation channels. They comprise 7 members, TRPC1–7, that all depend on phospholipid-hydrolysis (Storch et al., 2017). The TRPCs are activated by membrane receptors that couple via G_q/11_ and/or via G_i/o_ to isoforms of PLC. It was shown previously that TRPC1 plays an important role in store-operated calcium entry pathways, being activated by DAG and being inactivated by PKC-dependent phosphorylation (review: Ambudkar et al., 2017). Furthermore, DAG, as well as the membrane permeant DAG analogs OAG and DOG (1,2-dioctanoyl-cn-glycerol = DOG; 1-oleoyl-2-acetyl-sn-glycerol = OAG) activated TRPC2, −3, −6, and −7 ion channels apparently directly. The TRPC3, but not TRPC5 channels were directly opened by OAG, independent of PKC activation. Instead, OAG and DOG prevented TRPC5 and TRPC4 activation PKC-dependently (Venkatachalam et al., 2003). More recent studies (Storch et al., 2017) showed that TRPC4 and TRPC5 differ from the other TRPC channels, since they express a PDZ-binding motif in the C termini that also harbor a PKC phosphorylation site crucial for TRPC5 desensitization upon receptor activation (Zhu et al., 2005). Only if PKC-dependent phosphorylation of TRPC4/5 is prevented OAG or DOG were able to activate TRPC4/5 channels directly (Storch et al., 2017). Furthermore, while PIP_2_ depletion activated TRPC4 and −5 channels, it inhibited TRPC6 and −7 channels. In contrast to the directly DAG-dependently activated heteromultimers of TRPC3, TRPC6, and TRPC7 channel subunits, TRPC7 homomultimers show distinct properties such as constitutive activity and inhibition via PKC (Zhang and Trebak, 2014). Therefore, the DAG analogs OAG and DOG antagonistically affect various TRPC channels in mammals, either activating them directly, or inactivating them via PKC-dependent phosphorylation. We thus hypothesize that also in the hawkmoth DAG analogs antagonistically target TRPC-related ion channels that underlie the pheromone transduction cascade, since PLC and G_i/o_ were located to moth ORNs (review: Stengl, 2010).

So far, only cDNA encoding a TRPγ channel was identified in antennae of *Spodoptera littoralis*. Using *in situ* hybridization the transcript of this channel could be localized at the base and the shaft of pheromone-sensitive sensilla trichoidea hinting a role in olfactory transduction (Chouquet et al., 2009). Furthermore, a TRP channel was cloned and heterologously expressed from hawkmoth antennae (Ackermann, 2008) and DAG-dependent TRP-like channels were physiologically characterized from the hawkmoth (Krannich, 2008). How many TRP/TRPL-like ion channels are involved in the primary events of hawkmoth pheromone transduction and whether they are closed PKC-dependently as a form of short-term adaptation, however, is not known.

### We want to examine whether DAG-dependent ion channels underlie the phasic pheromone response

Since not much is known about DAG-dependent TRP channels in insect odor transduction (Zufall and Hatt, 1991; reviews: Fowler and Montell, 2013; Thiel et al., 2018) we wanted to examine whether DAG affects the primary events of pheromone transduction in the hawkmoth. Unfortunately, very specific ion channel antagonists for the different types of insect TRP channels are not known. Thus, to search for directly and indirectly DAG-dependent TRP channels in hawkmoths we employed the two membrane-permeable DAG analogs DOG and OAG that were shown before to affect TRP channels. *In vivo* in intact hawkmoths tip-recordings were performed. A brief pulse of bombykal at physiological concentrations elicits a sequence of potential changes in the bombykal-sensitive ORN that can be measured extracellularly (Nolte et al., 2016). Opening of bombykal-dependent ion channels in the cilium result in the depolarizing sensillum potential (Kaissling et al., 1987; review: Stengl, 2010). The depolarization elicits an action potential response in the axon with different kinetics. Only the first phasic action potential response within the first ~100 ms encodes bombykal concentration changes. About a second later, it is followed by the Orco-dependent late, long-lasting pheromone response that can persist over minutes or even hours after stimulation, depending on the strength of bombykal stimulation (Dolzer et al., 2003; Nolte et al., 2016). Thus, we examined whether perfusion of the two DAG analogs into the sensillum lymph affects the different parameters of the bombykal response. Since *M. sexta* ORNs are peripheral circadian pacemakers that modulate their pheromone sensitivity over the course of the day we performed our experiments either during the late activity phase at Zeitgebertime 1 (ZT 1), or during rest at ZT 9 (Schuckel et al., 2007; Flecke and Stengl, 2009; Flecke et al., 2010; Schendzielorz et al., 2012).

## Materials and methods

### Animals and preparation

For all experiments male *M. sexta* (Johannson) (Lepidoptera; Sphingidae) hawkmoths were used that were raised from egg at the University of Kassel. Larvae were fed on an artificial diet (modified after Bell and Joachim, 1976), adult moths could feed on sugar solution *ad libitum*. Animals were kept under long-day conditions (17 h:7 h, L:D) at 24–26°C and a relative humidity of 40–60%. Male pupae were isolated to avoid contact with female pheromone. For the recordings only adult male *M. sexta* that previously were kept at two dark-phases were caught 30 min before each experiment. Animals were fixed in a custom-built Teflon^TM^ holder and the flagellum of the right antenna was immobilized with dental wax (Boxing wax, Sybron/Kerr, Romulus, Michigan, USA). The upmost ~15 segments of the apical end of the antenna were cut off with micro-scissors. A glass electrode filled with hemolymph Ringer solution (6.4 mmol/L KCl, 12.0 mmol/L MgCl_2_, 1.0 mmol/L CaCl_2_, 12.0 mmol/L NaCl, 10 mmol/L HEPES, 354.0 mmol/L glucose-monohydrate) (Kaissling, 1995) was inserted into the lumen of the flagellum as indifferent electrode. To avoid desiccation the cut was sealed with electrode gel (*electrode gel*, GE Medical Systems Information Technologies, Freiburg, Germany). To access the sensillum lymph with forceps the upper quarter of the long trichoid sensilla from the apical row of the second remaining annulus were cut. The recording electrode filled with sensillum lymph Ringer solution (171.9 mmol/L KCl, 3.0 mmol/L MgCl_2_, 1.0 mmol/L CaCl_2_, 25 mmol/L NaCl, 10 mmol/L HEPES, 22.5 mmol/L glucose-monohydrate) (Kaissling, 1995) was pulled over one of the cut sensilla. To record the potentials between both electrodes Ag/AgCl wires were inserted into the Ringer solutions. The electrodes were connected to a custom-built amplifier (0 Hz−2 kHz, input impedance 10^12^Ω) where the recorded signals were amplified 200-fold. Subsequently, for data acquisition the signals were digitized with a Digidata 1200B (Molecular Devices, Sunnyvale, California, USA) and recorded with pCLAMP 8 software. All experiments were performed in the photophase of the animals. To investigate time-dependent differences in the pheromone responses one part of the recordings started at Zeitgebertime (ZT) 1, 1 h after lights on (ZT0), during the end of the activity phase of the animals. The other experiments started at ZT 9, during the middle of the resting phase. The recordings lasted for 2 h and all experiments were performed with room lights switched on.

### Application of drugs

Two membrane permeable DAG-analogs, 1,2-dioctanoyl-*sn*-glycerol (DOG) and 1-oleoyl-2-acetyl-*sn*-glycerol (OAG), were dissolved in dimethylsulfoxid (DMSO) and subsequently diluted in the sensillum lymph Ringer solution. For applications Ringer solutions containing concentrations of 1, 100 or 200 μmol l^−1^ DOG and OAG in 0.1% DMSO, were employed. Agents were applied passively via diffusion into the sensillum lymph (Kaissling et al., 1991) during the tip-recordings. All Ringer solutions were adjusted to pH 6.5. With mannitol osmolarity was adjusted to 475 mosmol l^−1^ for the hemolymph Ringer, and to 450 mosmol l^−1^ for the sensillum lymph Ringer.

### Pheromone stimulation

Pheromone was presented to the recording site as an airborne plume within a constant stream of air. The charcoal-filtered and moistened air was constantly blown over the preparation through a glass cartridge to ensure constant conditions throughout the recording. A second cartridge containing a filter paper (about 1 cm^2^) loaded with synthetic bombykal (E,Z-10,12-hexadecadienal) was installed parallel to the constant air stream. Stock solutions used for the experiments contained 10^−1^ mg/ml bombykal [generously provided by T. Christensen (University of Arizona, Tucson, Arizona, USA) and J. Krieger (University of Hohenheim, Stuttgart, Germany)] dissolved in *n*-hexane (Roth, Karlsruhe, Germany). The filter paper was loaded with 10 μl of the stock solutions resulting in final concentrations of 1 μg bombykal. For stimulation the air stream was re-directed to the second cartridge via a computer controlled valve (JFMH-5-PK-3, Festo, Esslingen, Germany). To avoid evaporation of the pheromone between stimulations a second valve (PA 202–004 P, Staiger, Erligheim, Germany) was positioned in front of the cartridge containing the bombykal. The outlets of both cartridges were placed at a distance of about 5 cm from the recording site. Every 5 min over a total recording time of 120 min 50 ms pheromone stimuli were applied, resulting in 24 stimulations per tip-recording. An interstimulus interval (ISTI) of 5 min was necessary to avoid adaptation of the bombykal response, as assessed by a decrease of the phasic pheromone response and a decline in the sensillum potential amplitude. Between recordings the cartridges containing bombykal were stored in scintillation vials at −20°C. They were used for about 10 recordings before replacing the filter paper.

### Acquisition protocols

Tip-recordings of spontaneous activity detected APs of two different amplitudes generated by the two ORNs innervating a trichoid sensillum while bombykal responses only activated one cell. Since AP amplitudes of the bombykal -sensitive ORN were always higher both ORNs could be distinguished easily (Dolzer et al., 2001). During stimulations not only the APs but also slower changes in the transepithelial potential (TEP) were recorded with glass electrodes. The TEP represents the sum of the membrane potentials of all cells located between the electrodes and does not only reflect the bombykal-dependent receptor potential generated. Thus, to prevent confusion with the receptor potential, it was termed sensillum potential (SP). The recorded pheromone responses covered a time frame of 5,161 ms with a pre-trigger part of 180 ms and a post-trigger part of 4,931 ms at a continuous sampling rate of 20 kHz (Clampex 8, episodic stimulation mode; Molecular Devices, Sunnyvale, California, USA).

### Data analysis and statistics

**Figure 1:**
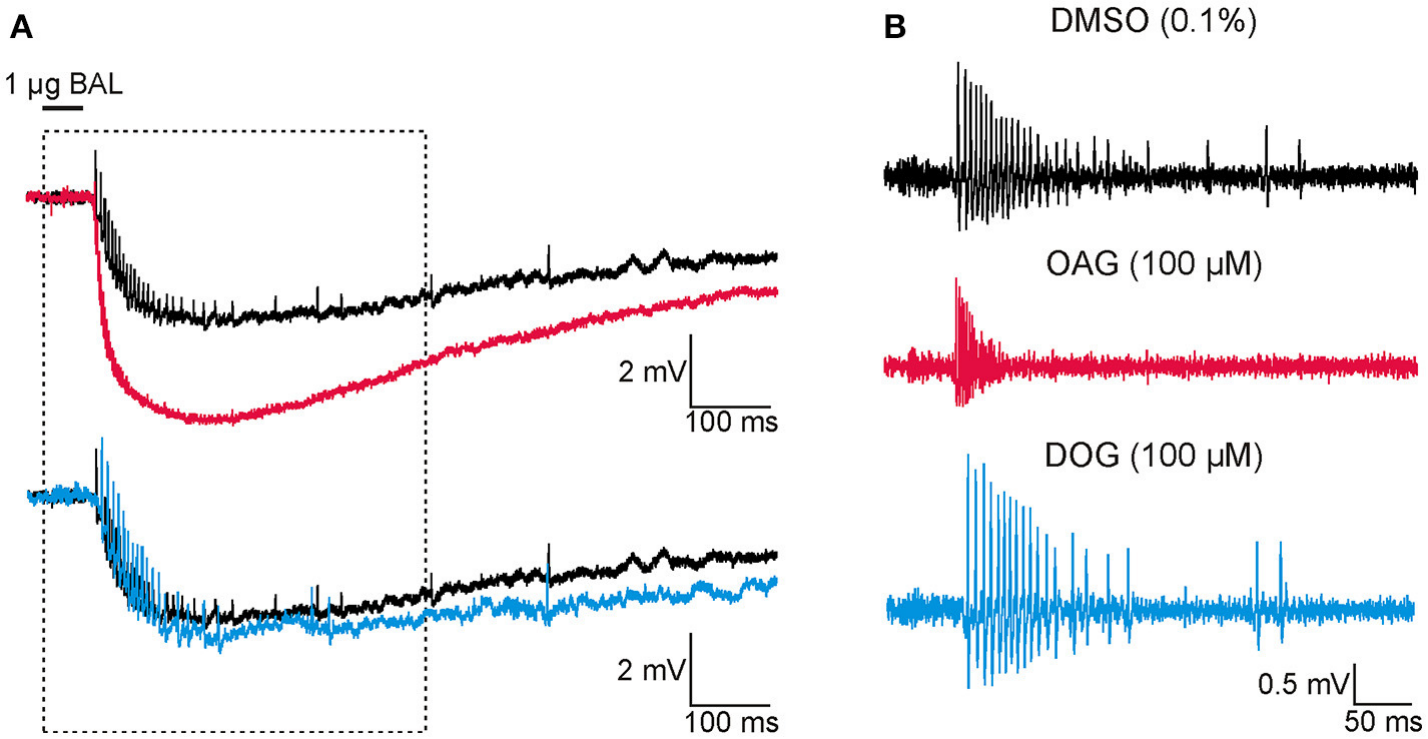
At Zeitgeberzeit 1 (ZT 1) the diacylglycerol-analog OAG increased the response to bombykal (BAL, 1 μg) while the diacylglycerol-analog DOG reduced it. Thus, both analogs target different DAG-dependent components of the BAL transduction cascade. Original tip-recordings from pheromone-sensitive trichoid sensilla on the antenna of the hawkmoth *Manduca sexta* show responses to 50 ms pulses of BAL (1 μg). Infusion of the DAG-analogs OAG (magenta, 100 μM) or DOG (cyan, 100 μM) via the tip-recording electrode were compared to control infusions with vehicle only (Ringer containing 0.1% DMSO, black) at the late activity phase at ZT 1. BAL-responses shown occurred about 30 min after the beginning of the recordings. **(A)** Unfiltered recordings. Infusion of OAG (magenta), but not of DOG (cyan) accelerated and increased the pheromone-dependent sensillum potential amplitude and action potential response. In contrast, DOG rather slowed down the repolarization of the pheromone-response. Recordings in boxed area were enlarged in **(B)**. **(B)** High-pass filtered BAL responses illustrated that OAG (magenta) increased the frequency of the BAL-dependent action potential response and rendered it more phasic, as compared to the control (black). In contrast, DOG (cyan) slightly decreased and slowed down the BAL-response.

The pheromone responses were analyzed using Spike 2 version 7.01 (Cambridge Electronic Design, Cambridge, UK; script written by A. Nolte) and Microsoft Excel. To analyze the SP the response was low-pass filtered at a cut-off frequency of 50 Hz. Evaluated was the maximal SP amplitude, that is the negative deflection of the TEP (Figure 1A) for each stimulation. Because of high variability between animals the SP amplitude of each recording was normalized to the first response. By comparing the normalized SP amplitude of the first 5 stimulations (beginning) and the amplitude of the last 5 stimulations (end) relative changes within recordings were analyzed. Additionally, the response was high-pass filtered with a cut-off frequency of 150 Hz to evaluate different parameters of the AP response. The peak frequency of the first 5 interspike intervals (AP frequency) evaluates the phasic pheromone response (Nolte et al., 2016). With post-stimulus time histograms (PSTHs; APs binned in intervals of 10 ms) the distribution of APs within the first 1,000 ms was analyzed, showing the phasic and tonic pheromone responses. For analysis of the late, long-lasting pheromone response the spikes occurring between 5 and 295 s after the bombykal stimulus were evaluated. Analysis of the spontaneous activity of ORNs without bombykal stimulation was performed equally. For statistical analysis results were tested for Gaussian distribution using the Kolmogorov-Smirnov test. If at least one data set failed the normality-test the Kruskal-Wallis test followed by Dunn's *post-hoc*-test was used for multiple comparisons. The Mann-Whitney test was applied to analyse the effects in relation to the recording time.

## Results

**Figure 2:**
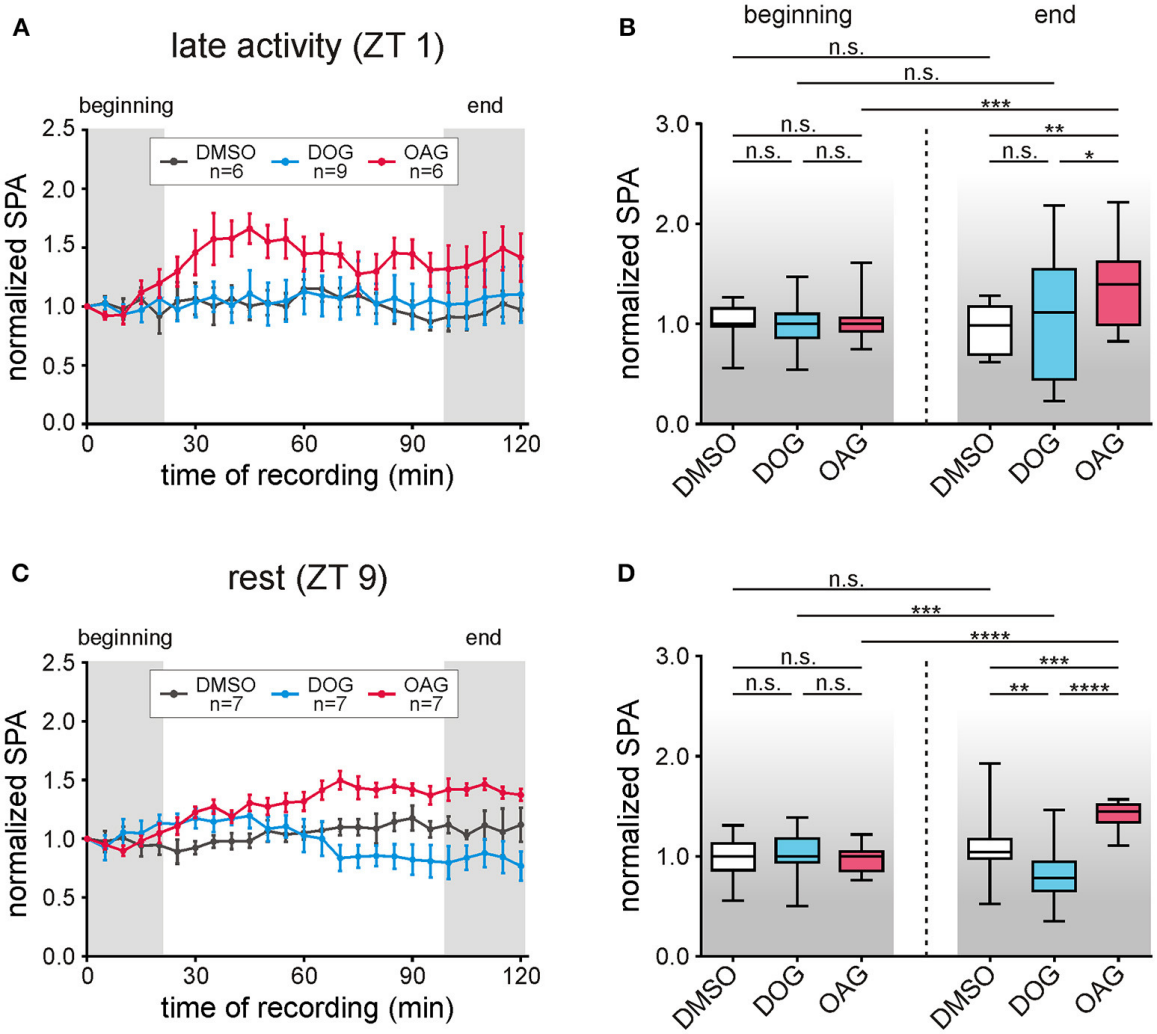
The DAG analog OAG (magenta) increased the bombykal (BAL)-dependent sensillum potential amplitude (SPA) at ZT 1 and ZT 9, while the DAG analog DOG (cyan) decreased it at ZT 9 only. Long-term tip-recordings over 120 min were performed from pheromone-sensitive trichoid sensilla on the antenna of the hawkmoth *Manduca sexta* either during the late activity phase at Zeitgebertime 1 (ZT 1) **(A,B)**, or during the resting phase at ZT 9 **(C,D)**. Every 5 min 50 ms pulses of BAL (1 μg) were applied. Via the tip-recording electrode either the DAG-analogs OAG (magenta, 100 μM) or DOG (cyan, 100 μM) were applied, in comparison to control infusions with vehicle only (Ringer containing 0.1% DMSO, black). The SPA in response to stimulation with BAL was normalized to the value of the first stimulation. Relative changes in the SPA were shown. Statistical analysis was performed during the first and last 20 min of the recordings (Table 1; gray areas **A,C)**. During the late activity phase (ZT 1–3) the normalized SPA was stable for control- and DOG recordings **(A,B)**. Application of 100 μM OAG increased the SPA over the course of the recording resulting in significant differences to controls and DOG. **(C,D)** Also, at ZT 9–11 the SPA remained stable in controls, while DOG decreased-, and OAG significantly increased it over time (**D**, exact *P*-values: Table 1). n.s. = not significant; ^*^P < 0.05; ^**^P < 0.01; ^***^P < 0.001; ^****^P < 0.0001; Mann-Whitney test or Kruskal-Wallis test with Dunn's *post-hoc* test for multiple comparison. For mean values ± SEM see S1.

**Figure 3:**
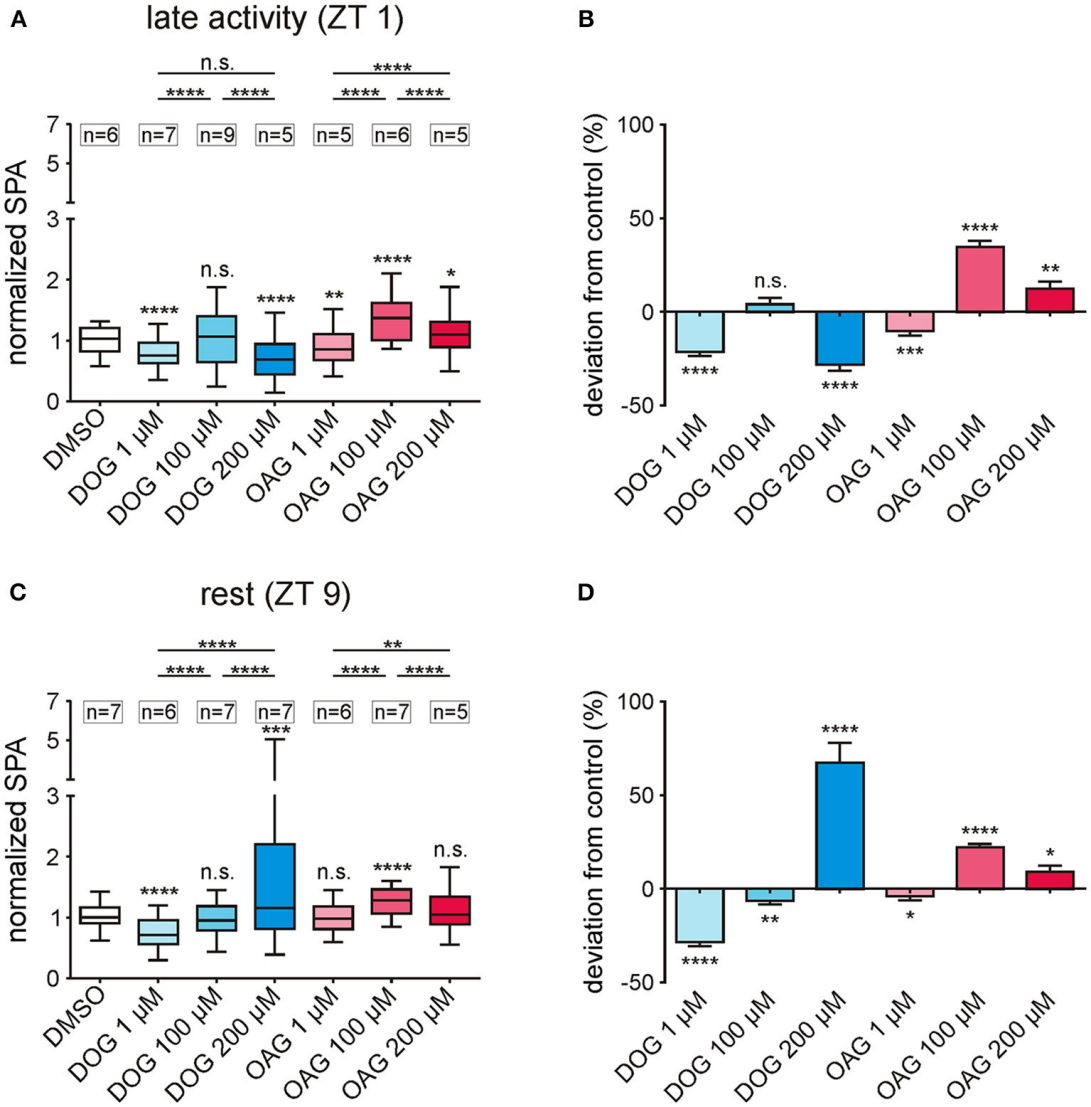
Infusion of different doses of the DAG analogs DOG and OAG showed that both analogs affected different ion channels underlying the bombykal-dependent normalized sensillum potential amplitude (SPA) with different sensitivity. **(A,B)** 1, 100, and 200 μM of the DAG-analogs DOG and OAG were applied in tip recordings during the late activity phase (ZT 1). The overall values **(A)** as well as the percentage changes to the controls **(B)** show a significant decrease in the normalized SPA in the presence of 1 μM (*n* = 7) or 200 μM DOG (*n* = 5), while 100 μM DOG (*n* = 9) had no effect. Application of 1 μM OAG reduced the normalized SPA (*n* = 5), while 200 μM OAG (*n* = 5) moderately- and 100 μM OAG more strongly increased it (*n* = 6). **(C)** During rest (ZT 9) the mean values of the normalized SPA were significantly reduced by 1 μM DOG (*n* = 6) and significantly increased by application of 200 μM DOG (*n* = 7) and 100 μM OAG (*n* = 7). All other concentrations showed no significant differences from the controls in their mean values. **(D)** However, all concentrations applied showed significant differences in the percentage change from the control (for exact *P*-values see Table 2; for mean values ± SEM see S2. n.s. = not significant; ^*^P < 0.05; ^**^P < 0.01; ^***^P < 0.001; ^****^P < 0.0001; Wilcoxon test, Mann-Whitney test or Kruskal-Wallis test with Dunn's *post-hoc* test for multiple comparison).

**Figure 4:**
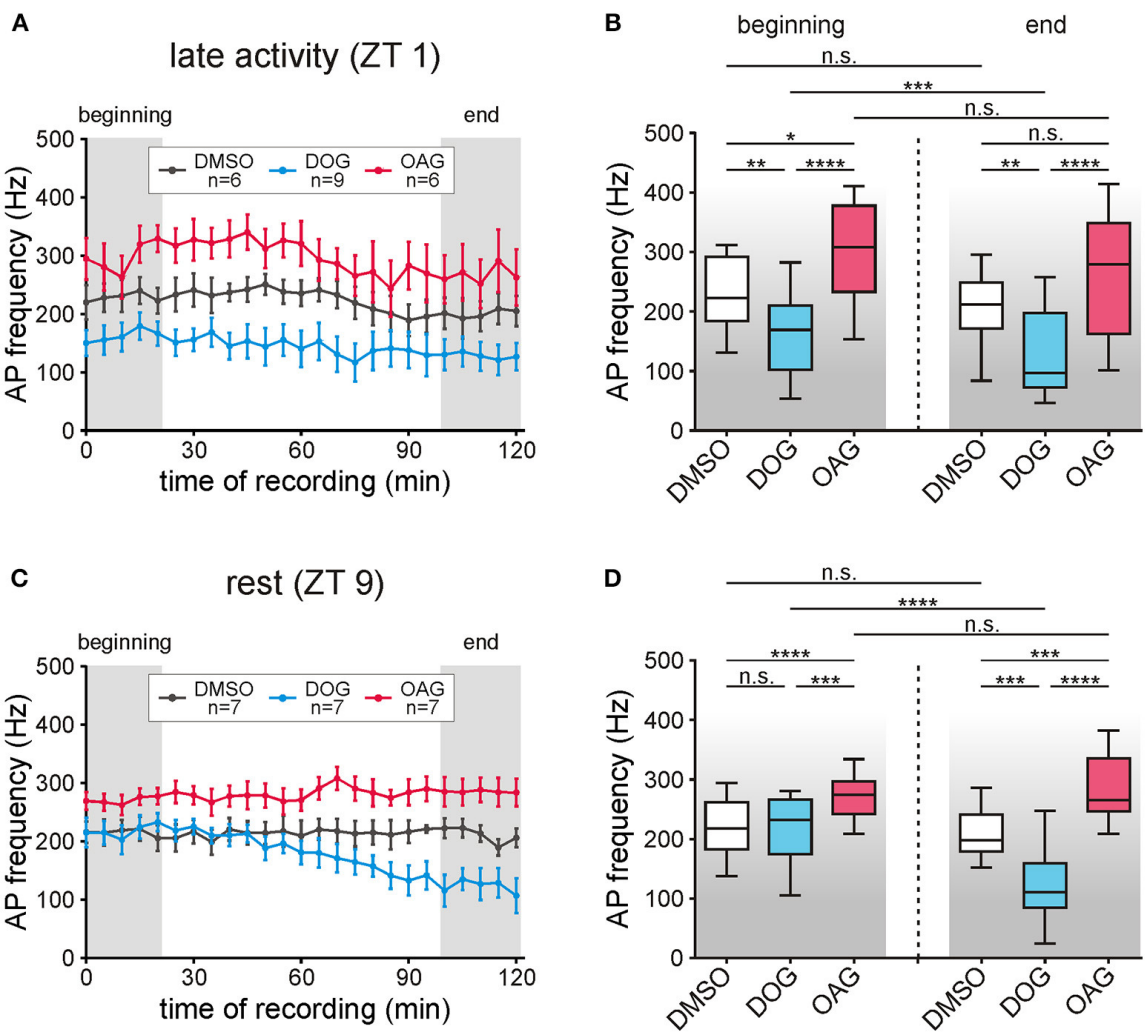
The phasic pheromone response was increased by OAG but decreased by DOG. **(A,B)** The phasic pheromone response (=frequency of the first 6 bombykal (BAL)-dependent action potentials (APs)) was stable in recordings during the late activity phase (ZT 1) for controls as well as for application of 100 μM OAG. However, with OAG phasic pheromone responses were significantly elevated in comparison to the controls at the beginning- and at the end of the recordings. Infusion of 100 μM DOG significantly reduced the phasic pheromone response as compared to controls already during the first 20 min and further decreased it toward the end of the recordings. **(C,D)** In the first hour of recordings during the animals' resting phase (ZT 9) application of 100 μM OAG significantly increased the AP frequencies while 100 μM DOG had no effect. In the second hour of the recordings DOG continuously decreased the frequency of the first 6 BAL-dependent APs while in controls as well as with 100 μM OAG the levels were unchanged (for exact *P*-values see Table 3; for mean values ± SEM see S3. n.s. = not significant; ^*^P < 0.05; ^**^P < 0.01; ^***^P < 0.001; ^****^P < 0.0001; Wilcoxon test, Mann-Whitney test or Kruskal-Wallis test with Dunn's *post-hoc* test for multiple comparison).

**Figure 5:**
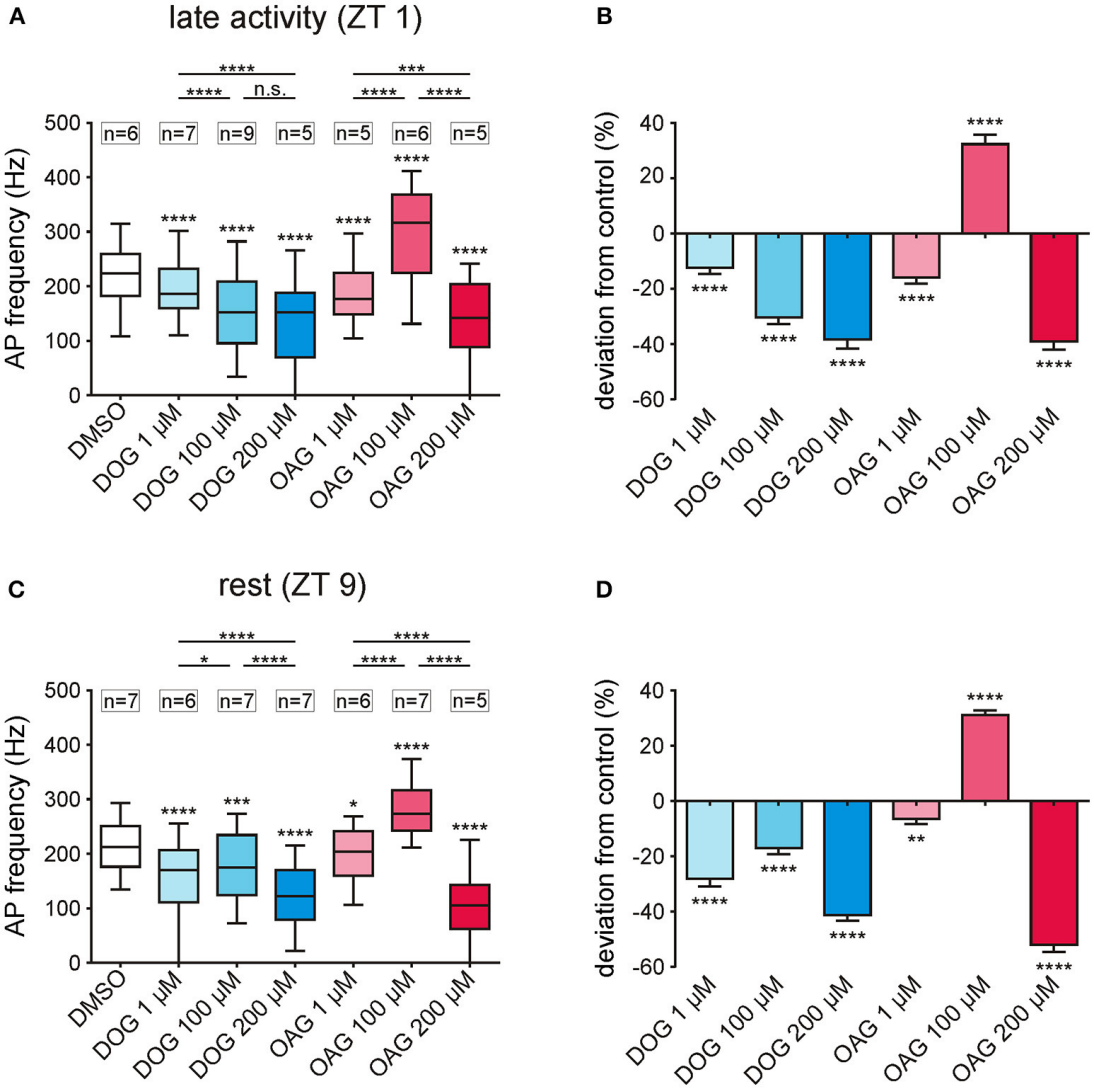
Only 100 μM OAG increased the phasic pheromone response while all other concentrations of both DAG-analogs caused reductions. **(A,C)** Box-plots show the phasic pheromone response [=frequency of the first 6 bombykal-dependent action potentials (APs)] over the 2 h recordings, with or without (control) infusion of different concentrations of DOG or OAG with 0.1% DMSO. A significant decrease in the phasic pheromone response was found for all concentrations of DOG (1, 100, 200 μM) as well as for 1 and 200 μM OAG at both Zeitgebertimes. Only application of 100 μM OAG increased the phasic pheromone response significantly during the late activity phase and at rest. **(B,D)** The percentage change illustrates that the highest concentrations of either DAG-analog resulted in the strongest reduction of the AP frequencies in comparison to the controls. Solely 100 μM OAG elevated the frequencies by about 30% in the late activity phase as well as during rest (for mean values ± SEM see S4; for exact *P*-values see S5; n.s. = not significant; ^*^P < 0.05; ^**^P < 0.01; ^***^P < 0.001; ^****^P < 0.0001; Wilcoxon test, Mann-Whitney test or Kruskal-Wallis test with Dunn's *post-hoc* test for multiple comparison).

**Figure 6:**
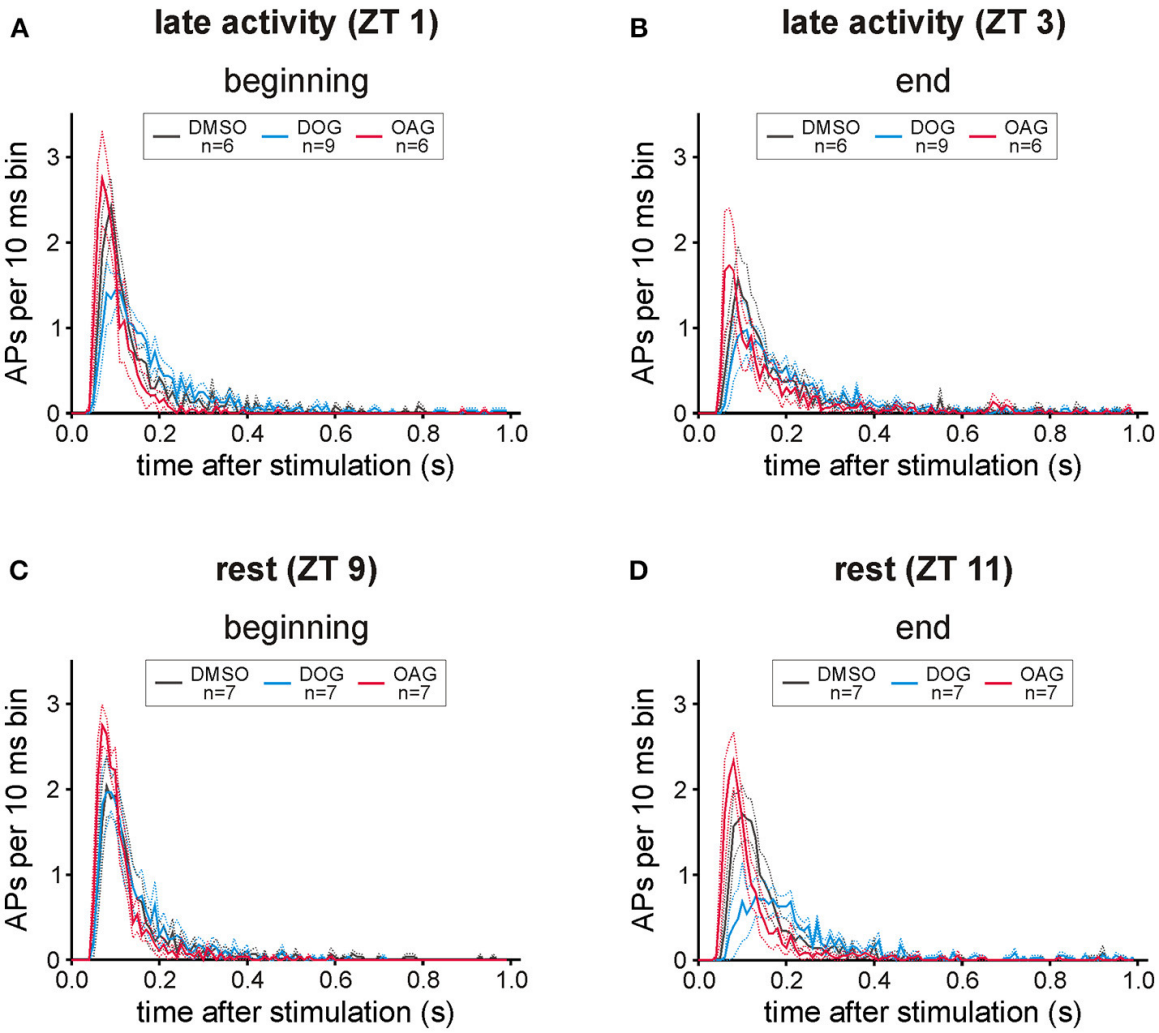
Both diacylglycerol-analogs affected the distribution of bombykal (BAL)- dependent action potentials (APs). In post-stimulus time histograms the mean number of APs in 10 ms bins was investigated for 1 s after BAL stimulation in the first- and last 20 min of each recording. **(A)** While in the first 20 min of recordings in the late activity phase (ZT 1) application of 100 μM OAG (magenta) only slightly increased and sped up the phasic-tonic response pattern, 100 μM DOG (cyan) reduced the number of APs per bin and rendered the response more tonic. **(B)** This shift to slower kinetics and an adapted AP response could be seen at the end of the recordings during the late activity phase (ZT 3), also in controls and with OAG, but strongest with DOG. **(C,D)** Also at rest (ZT 9) the pheromone responses were stronger and faster in the presence of OAG, as compared to controls and DOG infusion. Instead, application of DOG resulted in a strong reduction of APs per bin, which resulted in a decreased more tonic pheromone response especially at the end of recordings at ZT 11 (solid lines = mean; dotted lines = SEM).

**Figure 7:**
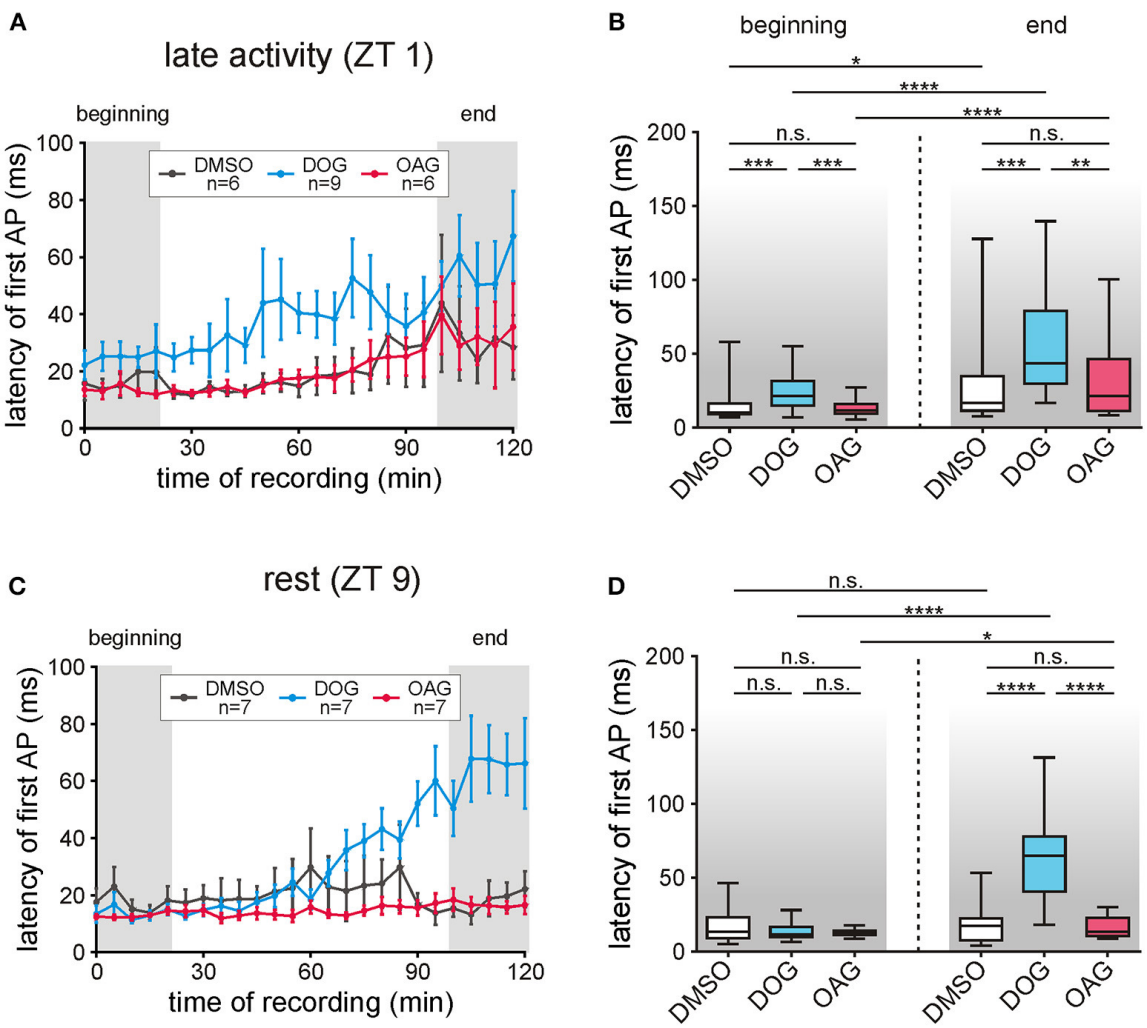
The latency of the first bombykal-dependent action potential (AP) increased only with DOG infusion. **(A,B)** During the late activity phase the latency of the first pheromone-dependent AP increased during the course of time in control recordings with 0.1% DMSO (*n* = 6) as well as with application of 100 μM DOG (*n* = 9) or 100 μM OAG (*n* = 6). In comparison to the controls only DOG significantly increased the latencies while application of OAG showed no significant differences. **(C,D)** In control recordings during rest (*n* = 7) no changes in the latency occurred during the course of the recordings and only a small increase was found under the influence of OAG (*n* = 7). However, application of DOG strongly and significantly increased the latency of the first AP in the last 20 min of the recordings (for exact *P*-values see Table 4; for mean values ± SEM see S6. n.s. = not significant; ^*^P < 0.05; ^**^P < 0.01; ^***^P < 0.001; ^****^P < 0.0001; Wilcoxon test, Mann-Whitney test or Kruskal-Wallis test with Dunn's *post-hoc* test for multiple comparison).

**Figure 8:**
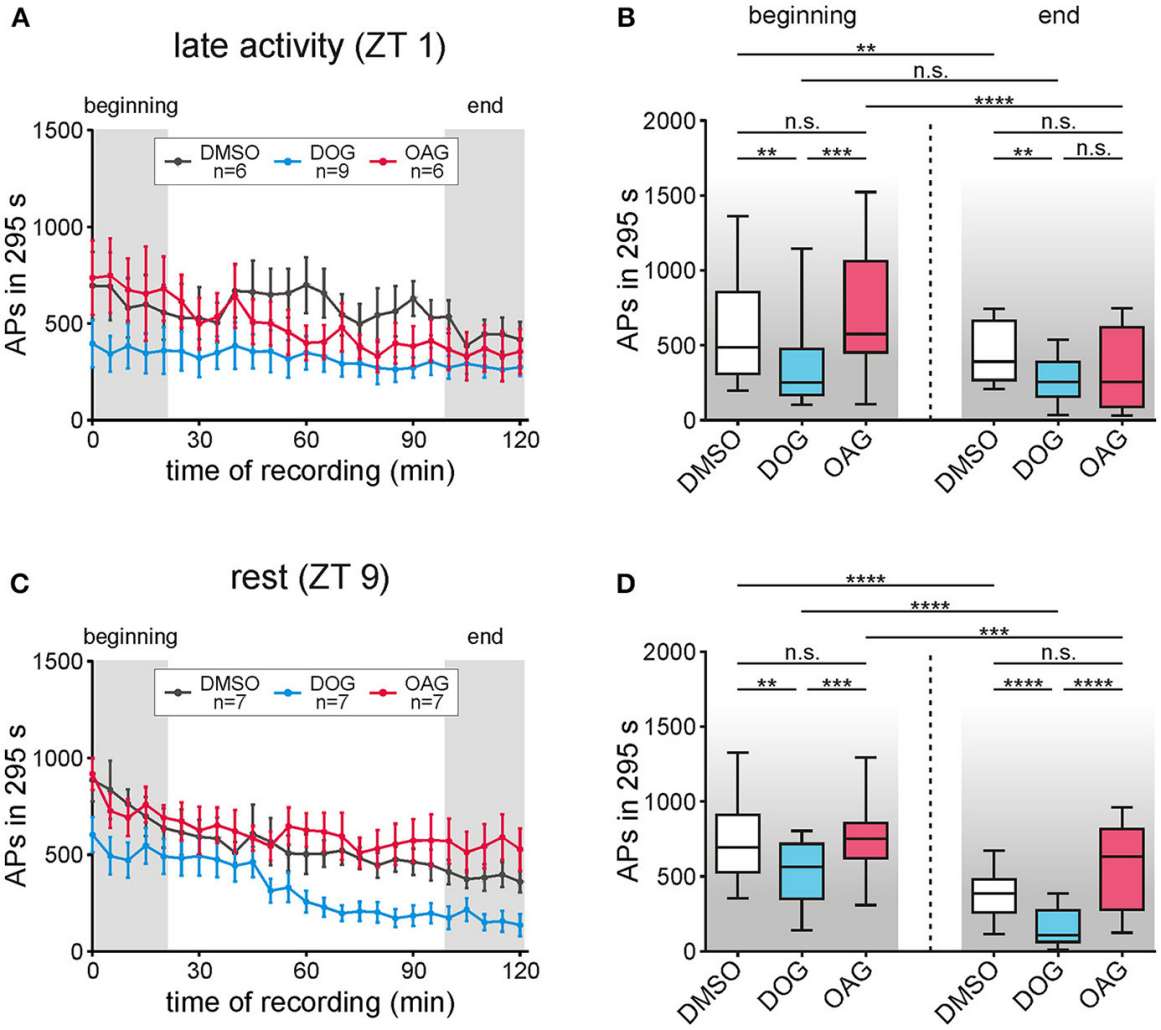
Only the diacylglycerol-analog DOG but not OAG decreased the late, long-lasting pheromone response (LLPR) at both Zeitgebertimes (ZTs). The number of APs between 5 s and 300 s after each stimulation with 1 μg bombykal was evaluated and statistical analysis was performed for the first and last 20 min of the recordings (gray areas, **A,C)**. **(A,B)** During the late activity phase the LLPR decreased in controls (*n* = 6) and during application of 100 μM OAG (*n* = 6) but no significant differences were found when comparing both groups. However, 100 μM DOG (*n* = 9) highly significantly decreased the LLPR in comparison to the controls but showed no changes over the course of time. **(C,D)** In recordings during rest the LLPR continuously decreased in controls (*n* = 7) as well as with DOG (*n* = 7) or OAG (*n* = 7). Again, application of OAG did not change the LLPR in comparison to the controls while with DOG a significant reduction and steady decline occurred in the first and the last 20 min of the recordings (for exact *P*-values see Table 5; for mean values ± SEM see S7. n.s. = not significant; ^*^P < 0.05; ^**^P < 0.01; ^***^P < 0.001; ^****^P < 0.0001; Wilcoxon test, Mann-Whitney test or Kruskal-Wallis test with Dunn's *post-hoc* test for multiple comparison).

**Figure 9:**
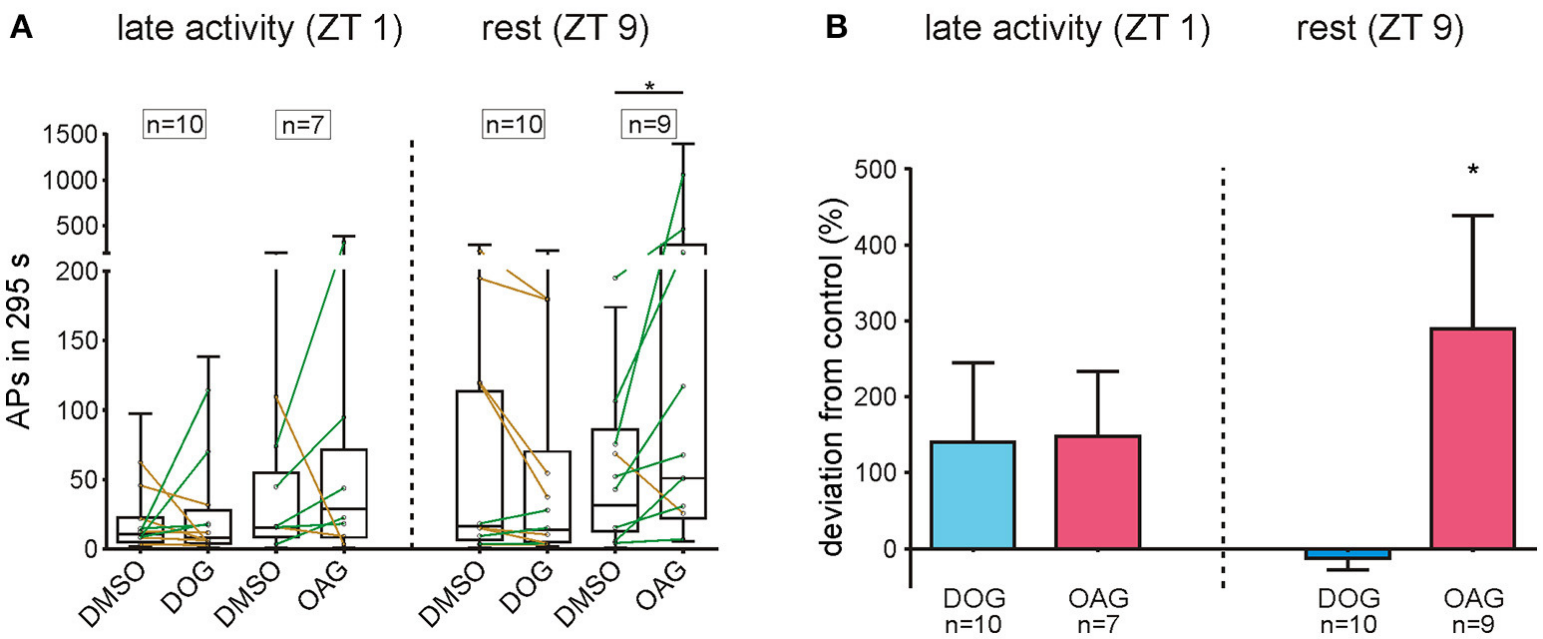
OAG significantly increased the spontaneous activity during rest. **(A)** The number of APs in 295 s intervals was measured under control conditions and with 100 μM DOG or OAG consecutively from the same sensillum. Mean values of single recordings are shown as connected dots and distribution of all values is represented as box plots. Both DAG-analogs either increased (green lines) or decreased (yellow lines) spontaneous activity in recordings at ZT 1 and ZT 9. Only during the resting phase application of 100 μM OAG (*n* = 9) significantly increased the spontaneous activity of olfactory receptor neurons. **(B)** The deviation from the respective control is shown as percentage change for each dataset. Although both DAG-analogs increased the spontaneous activity in the late activity phase this change was not significant. During rest application of DOG (*n* = 10) had no effect on the number of spontaneous APs while in the presence of OAG (*n* = 9) the deviation from controls was significant and the spontaneous activity increased by almost 300% (for exact *P*-values see Table 6; for mean values ± SEM see S8. n.s. = not significant; ^*^P < 0.05; ^**^P < 0.01; ^***^P < 0.001; ^****^P < 0.0001; Wilcoxon test).

In tip-recordings of bombykal-sensitive trichoid sensilla of the hawkmoth *Manduca sexta* we examined whether two membrane-permeable DAG-analogs, DOG and OAG that target TRP channels and PKC, affected different parameters of the bombykal responses (*n* = 91). Parameters examined were the bombykal-dependent sensillum potential amplitude (Figures 1A, 2, 3), the phasic bombykal-dependent action potential response (Figures 1B, 4–7), the Orco-dependent late, long-lasting pheromone response (Figure 8), and the spontaneous activity (Figure 9). The two DAG analogs were expected to either activate TRP channels directly and/or affect them indirectly/antagonistically via PKC activation. Activation of Ca^2+^-permeable TRP channels would increase the rise time and amplitude of the sensillum potential, as well as the frequency of the phasic pheromone response. In contrast, PKC inactivates TRP channels via negative feedback mechanisms at elevated intracellular Ca^2+^ concentrations. Thus, the rise time of the sensillum potential and its amplitude, as well as the phasic pheromone response would be decreased by PKC activation. Thus, we expected to observe antagonistic effects of both analogs if one preferentially activates TRP channels and the other preferentially activates PKC (Venkatachalam et al., 2003). The experiments were performed at the end of the activity phase (ZT 1–3) and during the resting phase (ZT 9–11) to account for circadian clock-dependent modulation of pheromone transduction in accordance with the physiological state of the animal. We wanted to know whether any of the two DAG-analogs directly or indirectly targets bombykal-dependent ion channels that are only available at rest or activity phases of the hawkmoth.

### Pheromone-dependent sensillum potential amplitudes were differentially and ZT-dependently affected by both DAG analogs (100 μM) in long-term tip-recordings

Two hrs long tip-recordings at ZT 1 and ZT 9 examined how infusion of 100 μM DOG (*n* = 16) and 100 μM OAG (*n* = 13) affected sensillum potentials in response to pheromone stimulation (50 ms pulses of 1 μg bombykal, every 5 min) (Figures 1A, 2A–D; Table 1; S1). With OAG present during the pheromone stimulations the sensillum potential amplitude rose faster to a higher amplitude and also declined faster (Figure 1A). The OAG effects were stronger and faster at ZT 1 (*n* = 6) compared to ZT 9 (*n* = 7) and became statistically significant during the course of the 2 hrs long recording (Figures 2A–D; Table 1; S1). In contrast, the presence of DOG had no significant effects on any parameter of the bombykal-dependent sensillum potential during ZT 1 (Figures 2A,B; Table 1), either because DOG-dependent ion channels were not available at the late activity phase, or, because they were already activated/inactivated. However, at ZT 9 during the course of the tip-recording DOG significantly reduced the sensillum potential amplitude (Figures 2C,D; Table 1; S1) and slowed down its rising- and declining phase (not shown). The amplitude of the control recordings remained unchanged during the course of the recordings at ZT 1 (*n* = 6) and ZT 9 (*n* = 7) (Figures 2A–D). Statistical analysis is summarized in Table 1, for mean values ± SEM (see Supplemental Figure S1). In summary, ZT-dependently OAG and DOG affected at least two different ion channels underlying the rising phase of the bombykal-dependent sensillum potential. Apparently, OAG opens a cation channel with fast kinetics that is available at both ZTs, but being more abundant or more available for activation at ZT1. In contrast DOG-dependent ion channels were not available at ZT 1, but appeared to underlie the pheromone-dependent sensillum potential at ZT 9. Directly or indirectly (via PKC) DOG either activated hyperpolarizing ion channels or inactivated depolarizing ion channels underlying the sensillum potential at ZT 9 but not at ZT 1.

**Table 1:** Statistical analysis of the normalized SPA in the presence or absence of 100 μM DOG or OAG during the late activity phase and at rest (Figure 2).

**Compared groups**		***P*****-value**		**Test**
		**Beginning (0–20 min)**	**End (100–120 min)**	
**LATE ACTIVITY (ZT 1)**				
DMSO	100 μM DOG	n.s. *P* > 0.9999	n.s. *P* > 0.9999	Kruskal-Wallis test with Dunn's post hoc test
DMSO	100 μM OAG	n.s. *P* > 0.9999	^**^P = 0.0042	
100 μM DOG	100 μM OAG	n.s. *P* > 0.9999	^*^P = 0.0210	
**Beginning vs. End**				
DMSO	DMSO	n.s. *P* = 0.8139		Mann-Whitney test
100 μM DOG	100 μM DOG	n.s. *P* = 0.6816		Mann-Whitney test
100 μM OAG	100 μM OAG	^***^P = 0.0008		Mann-Whitney test
**REST (ZT 9)**				
DMSO	100 μM DOG	n.s. *P* = 0.3910	^**^P = 0.0097	Kruskal-Wallis test with Dunn's post hoc test
DMSO	100 μM OAG	n.s. *P* > 0.9999	^***^P = 0.0002	
100 μM DOG	100 μM OAG	n.s. *P* = 0.2173	^****^P < 0.0001	
**Beginning vs. End**				
DMSO	DMSO	n.s. *P* = 0.2346		Mann-Whitney test
100 μM DOG	100 μM DOG	^***^P = 0.0002		Mann-Whitney test
100 μM OAG	100 μM OAG	^****^P < 0.0001		Mann-Whitney test

Level of significance: α = 0.05; n.s. = not significant;

* P < 0.05;

** P < 0.01;

*** P < 0.001;

**** *P < 0.0001*.

### Different doses of the DAG analogs DOG and OAG revealed that they each affected more than one ion channel underlying the bombykal-dependent sensillum potential

To further distinguish respectively targeted ion channels different doses of the two DAG analogs were applied (Figures 3A–D; Table 2; S2). We wanted to determine whether both analogs had dose-dependent effects on ion channels that generated the bombykal-dependent sensillum potential. The rational was that each analog has differential specificity for different TRP channels and for PKC. Thus, we expected that, e.g., OAG activates dose-dependently TRP channels and DOG activates dose-dependently PKC. Surprisingly, at both ZTs tested, none of the two components showed dose-dependent effects. Thus, each component either directly or indirectly affected the same ion channel antagonistically. Alternatively, they antagonistically affected more than one ion channel. Both analogs (OAG: *n* = 34; DOG: *n* = 40) could either increase or decrease the sensillum potential amplitude, depending on the concentrations and depending on the time of day (Figures 3A–D; Table 2; S2). Since the different doses of OAG (*n* = 34) showed the same distinct effects at both ZTs, just being more effective at ZT 1, the OAG-dependent ion channels were present and available at both ZTs, with higher abundance at ZT 1. At both ZTs 1 μM OAG decreased the sensillum potential amplitude (*n* = 11), higher concentrations increased it, with 100 μM being most effective (*n* = 13). Thus, at least 100 μM OAG are necessary to open a cation channel increasing the depolarization and apparently also the Ca^2+^ influx of the bombykal-dependent sensillum potential. Since 200 μM (*n* = 10) were less effective, an aversive effect appeared to accumulate at higher OAG concentrations, consistent with accumulating concentrations of intracellular Ca^2+^ or with PKC-dependent ion channel closure. At concentrations of 1 μM DOG (*n* = 13) had the same aversive effects as OAG (*n* = 11), but to a larger extent. In addition, DOG was more effective at ZT 9, in contrast to OAG. Since direction of effects and strength of effects were not similar at both ZTs for both agonists, they appeared to not affect the same targets. At 100 μM DOG had no effect at ZT 1 (*n* = 9). Slightly, but significantly 100 μM DOG reduced the bombykal-dependent sensillum potential amplitude at ZT 9 (*n* = 7). In contrast, at 200 μM DOG significantly reduced the bombykal-dependent sensillum potential amplitude at ZT 1 (*n* = 5), while it strongly increased it at ZT 9 (Figures 3A–D; Table 2; S2; *n* = 5). In summary, OAG and DOG targeted not the same ion channels. The OAG-dependent ion channels were more abundant at ZT 1, while the DOG-dependent ion channels were more abundant at ZT 9. Depending on the dose, the two analogs affected at least two different ion channels each. Or, they affected the same analog-dependent ion channel antagonistically at different concentrations. At 100 μM concentrations the effects of DOG and OAG differed most strongly. Thus, 100 μM concentrations of the analogs were suited best to distinguish different DAG-dependent targets in the hawkmoth's pheromone transduction cascade.

**Table 2:** Statistical analysis of the normalized SPA of long-term recordings in the presence or absence of 1, 100, or 200μM DOG or OAG during the late activity phase and at rest (Figure 3).

**Compared groups**		***P*****-value**		**Test**
		**Absolute values (a)**	**Deviation from control (%) (b)**	
**LATE ACTIVITY (ZT 1)**				
DMSO	1 μM DOG	^****^P < 0.0001	^****^P < 0.0001	(a): Kruskal-Wallis test with Dunn's post hoc test;
DMSO	100 μM DOG	n.s. *P* = 0.878	n.s. *P* = 0.3154	(b): Mann-Whitney test
DMSO	200 μM DOG	^****^P < 0.0001	^****^P < 0.0001	
1 μM DOG	100 μM DOG	^****^P < 0.0001		Kruskal-Wallis test with Dunn's post hoc test
1 μM DOG	200 μM DOG	n.s. *P* = 0.2918		
100 μM DOG	200 μM DOG	^****^P < 0.0001		
DMSO	1 μM OAG	^**^P = 0.0012	^***^P = 0.0001	(a): Kruskal-Wallis test with Dunn's post hoc test;
DMSO	100 μM OAG	^****^P < 0.0001	^****^P < 0.0001	(b): Mann-Whitney test
DMSO	200 μM OAG	^*^P = 0.0371	^**^P = 0.0023	
1 μM OAG	100 μM OAG	^****^P < 0.0001		Kruskal-Wallis test with Dunn's post hoc test
1 μM OAG	200 μM OAG	^****^P < 0.0001		
100 μM OAG	200 μM OAG	^****^P < 0.0001		
**REST (ZT 9)**				
DMSO	1 μM DOG	^****^P < 0.0001	^****^P < 0.0001	(a): Kruskal-Wallis test with Dunn's post hoc test;
DMSO	100 μM DOG	n.s. *P* = 0.3070	^**^P = 0.0076	(b): Mann-Whitney test
DMSO	200 μM DOG	^***^P = 0.0001	^****^P < 0.0001	
1 μM DOG	100 μM DOG	^****^P < 0.0001		Kruskal-Wallis test with Dunn's post hoc test
1 μM DOG	200 μM DOG	^****^P < 0.0001		
100 μM DOG	200 μM DOG	^****^P < 0.0001		
DMSO	1 μM OAG	n.s. *P* = 0.1184	^*^P = 0.0366	(a): Kruskal-Wallis test with Dunn's post hoc test;
DMSO	100 μM OAG	^****^P < 0.0001	^****^P < 0.0001	(b): Mann-Whitney test
DMSO	200 μM OAG	n.s. *P* = 0.0862	^*^P = 0.0389	
1 μM OAG	100 μM OAG	^****^P < 0.0001		Kruskal-Wallis test with Dunn's post hoc test
1 μM OAG	200 μM OAG	^**^P = 0.0042		
100 μM OAG	200 μM OAG	^****^P < 0.0001		

Level of significance: α = 0.05; n.s. = not significant;

* P < 0.05;

** P < 0.01;

*** P < 0.001;

**** *P < 0.0001*.

### OAG increased the phasic bombykal response

The pheromone-dependent depolarizing receptor potentials elicit the different kinetic phases of the action potential response. It has to be kept in mind, though, that the sensillum potential does not just reflect the receptor potentials (Dolzer et al., 2003). We focused on the phasic action potential response (= phasic pheromone response) since only the first few spikes of the pheromone response encode stimulus concentration (Dolzer et al., 2003). The phasic pheromone response in control recordings was stable over the 120 min long recordings at ZT 1 and ZT 9 (Figures 4A,C; *n* = 13). The median value of controls at the beginning of recordings during the activity phase (Table 3) was significantly higher than at the beginning of the resting phase (Table 3; S3). Application of 100 μM DOG significantly decreased the AP frequency compared to the controls during the activity phase, already at the beginning of the recording (Figures 4A,B; Table 3; S3). During rest this DOG-dependent decline occurred only at the end of the recordings (Figures 4C,D; Table 3; S3). In contrast, infusion of 100 μM OAG elevated the AP frequency slightly during the activity phase (Figures 4A,B). During rest (Figures 4C,D) application of OAG resulted in a highly significant increase of the AP frequency compared to the controls. In recordings with OAG (100 μM) at ZT 1 (Table 3; S3) and ZT 9 (Table 3; S3) there was no significant difference between the median values at the beginning and at the end of the recordings (Figures 4A–D). Only during application of DOG (100 μM) a significant decline between the first and the last 5 stimulations could be observed at ZT 1 as well as at ZT 9 (Table 3; S3). In summary, at both ZTs 100 μM OAG increased the frequency of the phasic pheromone response and also rendered it more phasic (Figure 1B). In contrast, 100 μM DOG decreased it faster at ZT 1 as compared to ZT 9. Strongest effects of both analogs occurred at the end of the recording during rest, at ZT 11. Thus, OAG- and DOG-dependent targets were available ZT-dependently.

**Table 3:** Statistical analysis of the AP frequency in the presence or absence of 100 μM DOG or OAG during the late activity phase and at rest (Figure 4).

**Compared groups**		***P*****-value**		**Test**
		**Beginning (0–20 min)**	**End (100–120 min)**	
**LATE ACTIVITY (ZT 1)**				
DMSO	100 μM DOG	^**^P = 0.0019	^**^P = 0.0018	Kruskal-Wallis test with Dunn's post hoc test
DMSO	100 μM OAG	^*^P = 0.0221	n.s. *P* = 0.1300	
100 μM DOG	100 μM OAG	^****^P < 0.0001	^****^P < 0.0001	
**Beginning vs. End**				
DMSO	DMSO	n.s. *P* = 0.4515		Mann-Whitney test
100 μM DOG	100 μM DOG	^***^P = 0.0005		Mann-Whitney test
100 μM OAG	100 μM OAG	n.s. *P* = 0.1349		Mann-Whitney test
**REST (ZT 9)**				
DMSO	100 μM DOG	n.s. *P* > 0.9999	^***^P = 0.0003	Kruskal-Wallis test with Dunn's post hoc test
DMSO	100 μM OAG	^****^P < 0.0001	^***^P = 0.0003	
100 μM DOG	100 μM OAG	^***^P = 0.0002	^****^P < 0.0001	
**Beginning vs. End**				
DMSO	DMSO	n.s. *P* = 0.5843		Mann-Whitney test
100 μM DOG	100 μM DOG	^****^P < 0.0001		Mann-Whitney test
100 μM OAG	100 μM OAG	n.s. *P* = 0.0708		Mann-Whitney test

Level of significance: α = 0.05; n.s. = not significant;

* P < 0.05;

** P < 0.01;

*** P < 0.001;

**** *P < 0.0001*.

### Only 100 μM OAG increased the phasic pheromone response at both ZTs, while all other doses tested of both OAG and Dog decreased it

At the late activity phase increasing doses of DOG (1-,100-, 200 μM) dose-dependently and significantly decreased the phasic pheromone response (Figures 5A,B; S4, 5; *n* = 21). At rest, however, the decreasing effect of 100 μM DOG was significantly smaller as compared to 1 and 200 μM (Figures 5C,D; S4, 5; *n* = 20). This indicated that at ZT 9, as compared to ZT 1 an additional target or ion channel was affected antagonistically by DOG. In contrast, at both ZTs 100 μM OAG (*n* = 13) increased the phasic pheromone response while at both ZTs all other concentrations tested decreased it (Figures 5A–D; S4, 5; *n* = 21). Thus, OAG had antagonistic effects either on the same or different ion channels. Furthermore, there was no direct correlation between DAG analog-dependent changes in the sensillum potential and the action potential response at both ZTs (Figures 3A–D, 5A–D). In summary, at both ZTs at least two different ion channels with antagonistic effects on the phasic pheromone response were affected by DOG and OAG, each. Furthermore, the sensillum potential clearly does not represent the receptor potential of the ORN since there were no direct correlations between both the bombykal-dependent sensillum potential and the bombykal-dependent action potential response.

### The distribution of action potentials revealed faster kinetics with OAG and slower with DOG

In post-stimulus time histograms (PSTHs) the distribution of bombykal-dependent APs occurring within the first 1000 ms after stimulation was analyzed (Figures 6A–D). During the course of the 2-h recordings in the activity phase (ZT 1) the response pattern of control recordings changed from phasic to more tonic responses (Figures 6A,B). Application of 100 μM DOG intensified this trend to tonic responses at the end of recordings during rest (Figures 6C,D) and directly from the beginning of recordings during the activity phase (Figures 6A,B). This became also apparent by the significantly reduced number of APs in the first 150 ms at the end of the resting phase (Figure 6D; *P* < 0.0001) and during the first 5 stimulations at the activity phase (Figure 6A; *P* < 0.0001). During the activity phase the distribution of APs under the influence of 100 μM OAG showed a similar pattern as the control recordings resulting in equal numbers of APs during the first 150 ms of the responses (Figure 6A). When OAG was applied at rest the phasic character of the response was maintained throughout the recordings (Figures 6C,D) and the number of APs during the first 150 ms was significantly increased compared to controls (Figures 6C,D; beginning: *P* < 0.0001, end: *P* = 0.0018). In summary, OAG rendered the pheromone-dependent action potential response more phasic increasing it at all ZTs while DOG rendered it less phasic ZT-dependently. DOG slowed it down and decreased it the most at the end of the long-term recordings at ZT 11.

### The latency of the first bombykal-dependent action potential was delayed by DOG but not by OAG

For temporal information processing the timing of the first stimulus-dependent action potential is most relevant. Thus, we analyzed how the DAG analogs affect the latency of the first bombykal-dependent action potential with respect to the start of the sensillum potential (Figures 7A–D; Table 4; S6). At all ZTs tested compared to vehicle controls (*n* = 13) OAG application (100 μM) did not significantly affect the latency of the pheromone response (*n* = 13) (Figures 7A–D; Table 4; S6). However, comparison of the overall recordings resulted in significantly lower latencies in the presence of OAG, decreasing the scatter of timing (*P* < 0.0001; Table 4; S6). In contrast, infusion of DOG increased the latency of the first pheromone-dependent spike at all ZTs (*n* = 16), but strongest at the end of the recording at rest, around ZT 11 (*n* = 7). In summary, while DOG increased the latency of the bombykal response ZT-dependently with the strongest effect at the end of the recording during rest, OAG rather decreased it.

**Table 4:** Statistical analysis of the latencies of the first bombykal-induced AP in the presence or absence of 100 μM DOG or OAG during the late activity phase and at rest (Figure 7).

**Compared groups**		***P*****-value**		**Test**
		**Beginning (0–20 min)**	**End (100–120 min)**	
**LATE ACTIVITY (ZT 1)**				
DMSO	100 μM DOG	^***^P = 0.0004	^***^P = 0.0004	Kruskal-Wallis test with Dunn's post hoc test
DMSO	100 μM OAG	n.s. *P* > 0.9999	n.s. *P* > 0.9999	
100 μM DOG	100 μM OAG	^***^P = 0.0008	^**^P = 0.0047	
**Beginning vs. End**				
DMSO	DMSO	^*^P = 0.0264		Mann-Whitney test
100 μM DOG	100 μM DOG	^****^P < 0.0001		Mann-Whitney test
100 μM OAG	100 μM OAG	^****^P < 0.0001		Mann-Whitney test
**REST (ZT 9)**				
DMSO	100 μM DOG	n.s. *P* > 0.9999	^****^P < 0.0001	Kruskal-Wallis test with Dunn's post hoc test
DMSO	100 μM OAG	n.s. *P* > 0.9999	n.s. *P* > 0.9999	
100 μM DOG	100 μM OAG	n.s. *P* > 0.9999	^****^P < 0.0001	
**Beginning vs. End**				
DMSO	DMSO	n.s. *P* = 0.2312		Mann-Whitney test
100 μM DOG	100 μM DOG	^****^P < 0.0001		Mann-Whitney test
100 μM OAG	100 μM OAG	^*^P = 0.0376		Mann-Whitney test

Level of significance: α = 0.05; n.s. = not significant;

* P < 0.05;

** P < 0.01;

*** P < 0.001;

**** *P < 0.0001*.

### Only DOG but not OAG decreased the late, long-lasting pheromone response

The late, long-lasting pheromone response occurs seconds to hours after the pheromone stimulus, not encoding stimulus concentration and duration, but rather being an hour-long reminder of the stimulus occurrence (reviews: Stengl, 2010; Stengl and Funk, 2013). Infusion of the DAG analog OAG had no significant effects on the late, long-lasting bombykal response compared to vehicle controls (*n* = 13) at all ZTs tested (*n* = 13) (Figures 8A–D; Table 5; S7). In contrast, application of DOG significantly decreased the late, long-lasting bombykal response at all ZTs tested (*n* = 16) (Figures 8A–D; Table 5; S7). The strongest effect of DOG occurred at the end of the recording at rest (Figures 8C,D; Table 5; S7), around ZT 11, where DOG almost abolished the late, long-lasting pheromone response (*n* = 7). In summary, while the DAG analog OAG had no effects, DOG significantly decreased the late, long-lasting pheromone response at both ZTs. At the end of the long-term tip-recordings at rest, DOG effects were strongest.

**Table 5:** Statistical analysis of the late, long-lasting pheromone response in the presence or absence of 100 μM DOG or OAG during the late activity phase and at rest (Figure 8).

**Compared groups**		***P*****-value**		**Test**
		**Beginning (0–20 min)**	**End (100–120 min)**	
**LATE ACTIVITY (ZT 1)**				
DMSO	100 μM DOG	^**^P = 0.0031	^**^P = 0.0040	Kruskal-Wallis test with Dunn's post hoc test
DMSO	100 μM OAG	n.s. *P* > 0.9999	n.s. *P* = 0.1857	
100 μM DOG	100 μM OAG	^***^P = 0.0003	n.s. *P* = 0.7280	
**Beginning vs. End**				
DMSO	DMSO	^**^P = 0.0047		Mann-Whitney test
100 μM DOG	100 μM DOG	n.s. *P* = 0.3670		Mann-Whitney test
100 μM OAG	100 μM OAG	^****^P < 0.0001		Mann-Whitney test
**REST (ZT 9)**				
DMSO	100 μM DOG	^**^P = 0.0032	^****^P < 0.0001	Kruskal-Wallis test with Dunn's post hoc test
DMSO	100 μM OAG	n.s. *P* > 0.9999	n.s. *P* = 0.3245	
100 μM DOG	100 μM OAG	^***^P = 0.0008	^****^P < 0.0001	
**Beginning vs. End**				
DMSO	DMSO	^****^P < 0.0001		Mann-Whitney test
100 μM DOG	100 μM DOG	^****^P < 0.0001		Mann-Whitney test
100 μM OAG	100 μM OAG	^***^P = 0.0008		Mann-Whitney test

Level of significance: α = 0.05; n.s. = not significant;

* P < 0.05;

** P < 0.01;

*** P < 0.001;

**** *P < 0.0001*.

### The DAG analogue OAG significantly increased spontaneous activity at rest

Insect ORNs can express spontaneous APs also in the absence of pheromone stimuli, however with quite strong variability. Thus, due to the wide scattering of values which appeared in all tip-recordings (Figure 9A) spontaneous activity was recorded for 40 min under control conditions with 0.1% DMSO, without application of pheromone pulses. After control recordings, the recording capillary was switched and the activity of the same sensillum was investigated for another 40 min in the presence of either 100 μM DOG or 100 μM OAG. The mean spontaneous activity of untreated ORNs ranged from 19.97 ± 6.04 APs at ZT 1 to 72.65 ± 27.14 APs in 295 s during rest (S8). Hence, the activity of ORNs is more than 10-fold increased after stimulation with 1 μg BAL (S7, 8). Application of 100 μM DOG either increased or decreased spontaneous activity (Figure 9A). In most cases only small changes in spontaneous activity were observed in the late activity phase (*n* = 10) as well as during rest (*n* = 10). In some recordings, especially when activity was low in controls during the activity phase an increased activity was measured in the presence of DOG (Figure 9A). With respect to controls DOG expressed non-significant increases at ZT 1 (Table 6) and during rest almost no changes occurred (Figure 9B; Table 6; S8). Infusion of 100 μM OAG almost always increased the spontaneous activity in comparison to the previous control (Figure 9B; Table 6; S8). During the late activity phase mean values for spontaneous activity were elevated with OAG application (*n* = 7) (S8). Spontaneous activity of some control recordings were higher at rest, nevertheless, here the OAG-dependent increases were even stronger (*n* = 9) (Table 6; S8). This increase was significant for the absolute values as well as for the percentage change (Table 6; S8). In summary, there was very strong scatter for absolute values of spontaneous activity at different ZTs recorded and OAG or DOG applications showed strongly variable, sometimes antagonistic responses. Thus, only at rest OAG significantly increased spontaneous activity, most likely via Ca^2+^-dependent effects.

**Table 6:** Statistical analysis of the spontaneous activity in the presence or absence of 100 μM DOG or OAG during the late activity phase and at rest (Figure 9).

**Compared groups**		***P*****-value**		**Test**
		**Absolute value (a)**	**Deviation from control (%) (b)**	
**LATE ACTIVITY (ZT 1)**				
DMSO (before DOG)	100 μM DOG	n.s. *P* > 0.9999	n.s. *P* = 0.8652	Wilcoxon test
DMSO (before OAG)	100 μM OAG	n.s. *P* = 0.3750	n.s. *P* = 0.1563	Wilcoxon test
DMSO (before DOG)	DMSO (before OAG)	n.s. *P* = 0.1609		Mann-Whitney test
100 μM DOG	100 μM OAG	n.s. *P* = 0.3130		Mann-Whitney test
**REST (ZT 9)**				
DMSO (before DOG)	100 μM DOG	n.s. *P* = 0.0840	n.s. *P* = 0.3750	Wilcoxon test
DMSO (before OAG)	100 μM OAG	^*^P = 0.0273	^*^P = 0.0195	Wilcoxon test
DMSO (before DOG)	DMSO (before OAG)	n.s. *P* = 0.8260		Mann-Whitney test
100 μM DOG	100 μM OAG	n.s. *P* = 0.0786		Mann-Whitney test

Level of significance: α = 0.05; n.s. = not significant;

* P < 0.05;

** P < 0.01;

*** P < 0.001;

**** *P < 0.0001*.

## Discussion

With tip-recordings of antennal trichoid sensilla *in vivo* it was examined whether two DAG analogs, DOG and OAG, modulated parameters of the primary events of bombykal transduction of male *M. sexta* hawkmoths. Recordings were performed at two different Zeitgebertimes (ZTs), because ORNs are circadian pacemaker neurons that regulate odor sensitivity in a daytime-dependent fashion. Since DAG has at least two different antagonistic functions, activation of Ca^2+^ permeable TRP ion channels and activation of PKC, we hoped to distinguish both functions with specific concentrations of DOG and OAG in hawkmoth pheromone transduction (review: Stengl, 2010). Indeed, at 100 μM concentrations the two analogs preferentially recognized different targets in the pheromone transduction cascade, daytime-dependently. While 100 μM OAG likely opened a Ca^2+^-permeable TRP-like channel underlying the pheromone-dependent receptor potential, DOG potentially closed pheromone-dependent channels very likely via PKC-dependent negative feedback. Thus, we hypothesize that OAG opens TRPC-type channels underlying pheromone transduction in *M. sexta*. In contrast, DOG activates PKC, and, thereby potentially closes TRPC-type ion channels which before were opened during the pheromone transduction cascade.

### The DAG analogs OAG and DOG appear not to affect orco in *M. sexta* ORNs

While it was suggested that insect odor transduction is mediated via an ionotropic mechanism based upon odor-gated activation of OR-Orco receptor ion channel complexes (Sato et al., 2008; Wicher et al., 2008), in hawkmoths previously we found no evidence for a role of the 7 transmembrane channel Orco in primary processes of pheromone transduction (Nolte et al., 2013, 2016). Instead, Orco appears to be a voltage-dependent ion channel that opens during the late, long-lasting pheromone response but not during the first few hundred ms of the phasic pheromone response. Since Orco agonists and antagonists were mostly effective during the activity phase of the hawkmoth, more Orco protein appears to be available during the night and early day as compared to the middle of the day. In addition, Orco constitutes a spontaneously opening Ca^2+^-permeable non-specific cation channel with a reversal potential around 0 mV. Thus, Orco depolarizes the membrane potential, thereby controlling spontaneous activity of hawkmoth and fruitfly ORNs. Furthermore, Orco appears to be opened voltage-, and second messenger-dependently (Larsson et al., 2004; Benton et al., 2007; Sato et al., 2008; Wicher et al., 2008; Deng et al., 2011; Jones et al., 2011; Sargsyan et al., 2011; Nolte et al., 2013, 2016). In addition to the primary ionotropic odor transduction, Wicher et al. (2008) suggested an additional slower transduction via OR coupling to G_αs_ resulting in elevation of cAMP levels. In *D. melanogaster* Orco needs to be phosphorylated PKC-dependently first, before it can be activated by cGMP and cAMP (Sargsyan et al., 2011). Thus, OAG, as well as phorbol ester (PMA) can activate Orco channels PKC-dependently in fruitflies. However, it is unlikely that in our experiments OAG or DOG activated Orco. Since in the hawkmoth OAG did not affect the late, long-lasting pheromone response at ZT 1 and ZT 9, it can be assumed that OAG does not affect Orco. In addition, since DOG decreased the late, long-lasting pheromone response at both ZTs with strongest effect at ZT 11, it is also unlikely that it mediated its effect via Orco activation or Orco inactivation since more Orco protein is present during the activity phase of the hawkmoth. Furthermore, also the OAG-dependent activation of spontaneous activity which only occurred at ZT 9 cannot predominantly be mediated via Orco, since apparently Orco abundance at ZT 1 is higher as compared to ZT 9 (Nolte et al., 2013, 2016). Therefore, we suggest that additional TRP-like ion channels are activated during the late, long-lasting pheromone response and that Ca^2+^-dependent ion channels such as TRP-like ion channels are also involved in the generation of spontaneous activity of ORNs, next to Orco. Since our pharmacological studies cannot exclude additional effects on so far unknown targets, molecular identification and manipulation of Orco and TRP-like ion channels in the hawkmoth are necessary to further characterize pheromone transduction cascades. So far, our studies do support a role of Orco as pacemaker channel in hawkmoth ORNs that controls the resting membrane potential, but not as the primary channel of pheromone transduction. Future studies need to separate different possible functions of Orco in different insect species as chaperon that locates and maintains ORs in ciliary membranes, as pacemaker channel affecting membrane potential, and as odor receptor-ion channel complex during the primary events of transduction.

### Pheromone transduction in hawkmoth ORNs involves phospholipase c-dependent signaling cascades activating several ion channels with properties of TRP-type channels

Biochemical studies demonstrated that pheromone application to cockroach and moth antennae caused rapid and transient rises in IP_3_ levels (Boekhoff et al., 1990, 1993; Breer et al., 1990). Thus, activation of pheromone receptors activates PLC G-protein dependently in insect antennae. Pheromone application to hawkmoth ORNs in primary cell culture elicited a stereotyped sequence of directly and indirectly Ca^2+^ dependent inward currents. The first pheromone-dependent current promoted Ca^2+^ influx at positive potentials. This very transient pheromone-dependent Ca^2+^ channel was blocked by high concentrations of extracellular Ca^2+^ within ms. Also, inclusion of IP_3_ in the patch pipette directly or indirectly opened a very transient Ca^2+^ channel that was blocked by Ca^2+^ influx within ms in hawkmoth ORNs (Stengl, 1994; review: Stengl, 2010). Since the IP_3_-dependent Ca^2+^ current mimicked the pheromone-dependent Ca^2+^ current also in hawkmoths bombykal receptors appear to couple to PLC. While it was demonstrated that this first pheromone-dependent Ca^2+^ channel cannot be identical to the unspecific cation channel Orco, however, its molecular identity remains unknown (Stengl, 1994; Nolte et al., 2013, 2016). Since different TRPC channels were shown to interact with IP_3_ receptors that are located to the endoplasmic reticulum (review: Ong et al., 2014), it is possible, that this first pheromone-dependent Ca^2+^ channel belongs to TRPC-like channels. Future studies need to test this hypothesis further and need to examine whether IP_3_ receptor activation is obligatory for bombykal responses in *M. sexta in vivo*.

Next, in hawkmoth ORNs the pheromone-dependent influx of Ca^2+^ elicited a Ca^2+^-dependent inward current with linear I/V curve and reversal potential around 0 mV indicative of an unspecific cation current. It expressed bimodal dependence on extracellular Ca^2+^. Increases of extracellular Ca^2+^ first activated, and, then, within seconds blocked this second pheromone-dependent inward current (Stengl, 1993). While the molecular identity of its underlying pheromone-dependent ion channel is not known, again its properties resemble properties of TRP-type ion channels (review: Nilius and Flockerzi, 2014). The third pheromone-dependent inward current was also a nonspecific cation current with reversal potential around 0 mV. However, it was less Ca^2+^ selective than the second pheromone-dependent current and was activated by PKC. While PKC closed previously opened pheromone-dependent TRP-like ion channels, during this later time window after pheromone application, it opened PKC-dependent cation channels that were less Ca^2+^-permeable (Stengl, 1993, 1994; Dolzer et al., 2008). Finally, strong or long pheromone stimuli elevate cGMP levels in ORNs that appeared to govern cGMP-dependent ion channel activity and closed other pheromone-dependent ion channels which were opened previously. The cGMP-dependent channels expressed much slower kinetics compared to the pheromone-activated Ca^2+^- or PKC-dependent ion channels and allowed for bombykal responses under conditions of long-term adaptation (Ziegelberger et al., 1990; Boekhoff et al., 1993; Stengl et al., 2001; Dolzer et al., 2003; Flecke et al., 2006; Krannich and Stengl, 2008). Voltage- and Ca^2+^- dependent K^+^ - and Cl^−^ channels then repolarized the ORNs (review: Stengl, 2010).

These different PLC- and pheromone-dependent ion channels in hawkmoth ORNs resembled properties of TRP/TRPL-type channels most closely. Our experiments with the DAG analogs are in accordance with our hypothesis that bombykal receptors couple to PLC and gate TRP-type ion channels. The non-linear, not dose-dependent but ZT-dependent effects of both, OAG and DOG, on the sensillum potential amplitude and the phasic action potential response indicated that several TRP-like ion channels may be involved in bombykal transduction in the hawkmoth *M. sexta*. We hypothesize that 1 μM of DOG and OAG inhibited DAG-dependent TRPC ion channels such as channels resembling TRPC4 and TRPC5 (Storch et al., 2017). At 100 μM DOG apparently additional DAG-dependent channels resembling TRP2, −3, −6, or −7 were activated that were more available at ZT 9 as compared to ZT 1. Also, OAG at 1 μM concentrations inhibited channels resembling TRPC4 and TRPC5, but less effectively as compared to DOG. But at 100 μM concentrations OAG activated at least one TRPC2, −3, −6, or −7- like channel much more effectively compared to DOG. This OAG-dependent channel was more available at ZT 1 as compared to ZT 9. Further increase of the dose of OAG apparently caused desensitization of the OAG-dependent TRPC-like ion channels similar to the PKC-dependent TRPC7-desensitization (Zhang and Trebak, 2014). We do not know yet which and how many TRP-type ion channels are expressed in ORNs of the hawkmoth. However, since we could elicit a sequence of at least three different bombykal-dependent inward currents upon G-protein activation or after application of IP_3_ (Stengl, 1993, 1994), we expect at least 3 different TRPC-type ion channels that even may cooperate with IP_3_ dependent ion channels. The modulation of the different parameters of the bombykal response by 100 μM OAG *in vivo* is most consistently explained via OAG-dependent activation of a strongly Ca^2+^ permeable TRP-type ion channel. This OAG-dependent TRP channel can be blocked either via PKC or Ca^2+^/calmodulin, since increasing concentrations of OAG decreased the sensillum potential amplitude and the phasic action potential response. In addition, 100 μM DOG effects are consistent with DOG-dependent activation of PKC and PKC-dependent closure of TRP-type ion channels activated by the pheromone transduction cascade. In the mammalian vomeronasal organ the DAG-dependently activated TRPC2 channel is the primary ion channel underlying pheromone transduction (Lucas et al., 2003). In addition, it was shown that IP_3_ receptor gating appeared not to be involved in TRPC2 activation (Chamero et al., 2017). Whether this is also true for *M. sexta* pheromone transduction and whether/how many TRP channels underly bombykal transduction remains to be examined.

## Author contributions

PG performed all experiments, evaluated the data with statistics, prepared all Figures, and contributed to the final version of the manuscript. MS wrote the manuscript and contributed to the interpretation of the data. MS obtained finances for the project with DFG grants.

### Conflict of interest statement

The authors declare that the research was conducted in the absence of any commercial or financial relationships that could be construed as a potential conflict of interest.
