# Supplementary material for: The Diacylglycerol Analogs OAG and DOG Differentially Affect Primary Events of Pheromone Transduction in the Hawkmoth Manduca sexta in a Zeitgebertime-Dependent Manner Apparently Targeting TRP Channels
Source: Front Cell Neurosci. 2018 Jul 24;12:218. doi: 10.3389/fncel.2018.00218 (PMC6066562; doi:10.3389/fncel.2018.00218)
Supplement: Supplementary file 1 [file Data_Sheet_1.docx]

Supplementary Material

**S. 1.** Mean values ± std. error of the normalized SPA (Fig. 2) during the first and last 20 min of recordings in the late activity phase and at rest in the presence or absence of different DAG-analogs.

| **Group** | **Late activity (ZT 1)** | | | **Rest (ZT 9)** | | |
| --- | --- | --- | --- | --- | --- | --- |
|  | n | beginning  (0-20 min) | end  (100-120 min) | n | beginning  (0-20 min) | end  (100-120 min) |
| DMSO | 6 | 1.001 ± 0.037 | 0.954 ± 0.045 | 7 | 0.974 ± 0.035 | 1.090 ± 0.057 |
| 100 µM DOG | 9 | 0.999 ± 0.037 | 1.064 ± 0.096 | 7 | 1.032 ± 0.039 | 0.825 ± 0.053 |
| 100 µM OAG | 6 | 1.036 ± 0.039 | 1.392 ± 0.082 | 7 | 0.975 ± 0.022 | 1.413 ± 0.025 |

**S. 2.** Mean values ± std. error of the normalized SPA in long-term tip recordings in the presence or absence of different concentrations of the DAG-analogs DOG and OAG (Fig. 3). Additionally the values of the percentage changes to control recordings from both recordings times, the late activity phase and rest, are shown.

| **Group** | **Late activity (ZT 1)** | | | **Rest (ZT 9)** | | |
| --- | --- | --- | --- | --- | --- | --- |
|  | n | absolute values | Deviation from control (%) | n | absolute values | Deviation from control (%) |
| DMSO | 6 | 1.008 ± 0.020 |  | 7 | 1.038 ± 0.018 |  |
| 1 µM DOG | 7 | 0.793 ± 0.023 | -21.38 ± 2.23 | 6 | 0.743 ± 0.023 | -28.40 ± 2.24 |
| 100 µM DOG | 9 | 1.048 ± 0.033 | +4.01 ± 3.31 | 7 | 0.973 ± 0.023 | -6.27 ± 2.18 |
| 200 µM DOG | 5 | 0.725 ± 0.034 | -28.03 ± 3.41 | 7 | 1.738 ± 0.179 | +67.45 ± 10.52 |
| 1 µM OAG | 5 | 0.907 ± 0.038 | -10.06 ± 2.73 | 6 | 0.996 ± 0.024 | -4.04 ± 2.05 |
| 100 µM OAG | 6 | 1.358 ± 0.032 | +34.70 ± 3.15 | 7 | 1.267 ± 0.019 | +22.11 ± 1.80 |
| 200 µM OAG | 5 | 1.132 ± 0.038 | +12.33 ± 3.75 | 5 | 1.132 ± 0.033 | +9.05 ± 3.19 |

**S. 3.** Mean values ± std. error of the AP frequency (Fig. 4) during the first and last 20 min of recordings in the late activity phase and at rest in the presence or absence of different DAG-analogs.

| **Group** | **Late activity (ZT 1)** | | | **Rest (ZT 9)** | | |
| --- | --- | --- | --- | --- | --- | --- |
|  | n | beginning  (0-20 min) | end  (100-120 min) | n | beginning  (0-20 min) | end  (100-120 min) |
| DMSO | 6 | 228.5 ± 10.69 | 201.0 ± 11.53 | 7 | 215.5 ± 8.52 | 210.4 ± 6.76 |
| 100 µM DOG | 9 | 162.4 ± 9.99 | 128.3 ± 10.80 | 7 | 218.3 ± 9.18 | 122.8 ± 11.07 |
| 100 µM OAG | 6 | 298.6 ± 14.68 | 267.2 ± 19.66 | 7 | 270.7 ± 6.40 | 285.3 ± 9.84 |

**S. 4.** Mean values ± std. error of the AP frequencies in long-term tip recordings in the presence or absence of different concentrations of the DAG-analogs DOG and OAG (Fig. 5). Additionally the values of the percentage changes to control recordings from both recordings times, the late activity phase and rest, are shown.

| **Group** | **Late activity (ZT 1)** | | | **Rest (ZT 9)** | | |
| --- | --- | --- | --- | --- | --- | --- |
|  | n | absolute values (Hz) | Deviation from control (%) | n | absolute values (Hz) | Deviation from control (%) |
| DMSO | 6 | 222.0 ± 4.91 |  | 7 | 213.8 ± 3.79 |  |
| 1 µM DOG | 7 | 194.5 ± 4.88 | -12.39 ± 2.20 | 6 | 153.8 ± 5.99 | -28.07 ± 2.80 |
| 100 µM DOG | 9 | 154.7 ± 5.40 | -30.33 ± 2.43 | 7 | 177.6 ± 4.99 | -16.92 ± 2.34 |
| 200 µM DOG | 5 | 137.0 ± 7.39 | -38.29 ± 3.33 | 7 | 125.6 ± 4.44 | -41.27 ± 2.08 |
| 1 µM OAG | 5 | 186.7 ± 5.05 | -15.92 ± 2.28 | 6 | 200.1 ± 4.23 | -6.39 ± 1.98 |
| 100 µM OAG | 6 | 293.8 ± 7.42 | +32.33 ± 3.34 | 7 | 280.1 ± 3.78 | +31.02 ± 1.77 |
| 200 µM OAG | 5 | 135.4 ± 6.85 | -39.03 ± 3.09 | 5 | 102.9 ± 5.61 | -52.07 ± 2.61 |

**S. 5.** Statistical analysis of the AP frequencies of long-term recordings in the presence or absence of 1, 100, or 200 µM DOG or OAG during the late activity phase and at rest (Fig. 5). Level of significance: α= 0.05; n.s. = not significant; ***P<0.05; ****P<0.01; *****P<0.001; ****P<0.0001

| **Late activity (ZT 1)** | | | | |
| --- | --- | --- | --- | --- |
| **Compared groups** | | **P-Value** | | **Test** |
|  |  | Absolute value (a) | Deviation from control (%) (b) |  |
| DMSO | 1 µM DOG | **** P< 0.0001 | **** P< 0.0001 | (a): Kruskal-Wallis test with Dunn’s post hoc test; (b): Mann-Whitney test |
| DMSO | 100 µM DOG | **** P< 0.0001 | **** P< 0.0001 |  |
| DMSO | 200 µM DOG | **** P< 0.0001 | **** P< 0.0001 |  |
| 1 µM DOG | 100 µM DOG | **** P< 0.0001 |  | Kruskal-Wallis test with Dunn’s post hoc test |
| 1 µM DOG | 200 µM DOG | **** P< 0.0001 |  |  |
| 100 µM DOG | 200 µM DOG | n.s. P= 0.2938 |  |  |
| DMSO | 1 µM OAG | **** P< 0.0001 | **** P< 0.0001 | (a): Kruskal-Wallis test with Dunn’s post hoc test; (b): Mann-Whitney test |
| DMSO | 100 µM OAG | **** P< 0.0001 | **** P< 0.0001 |  |
| DMSO | 200 µM OAG | **** P< 0.0001 | **** P< 0.0001 |  |
| 1 µM OAG | 100 µM OAG | **** P< 0.0001 |  | Kruskal-Wallis test with Dunn’s post hoc test |
| 1 µM OAG | 200 µM OAG | *** P= 0.0004 |  |  |
| 100 µM OAG | 200 µM OAG | **** P< 0.0001 |  |  |
| **Rest (ZT 9)** | | | | |
|  | | Absolute value (a) | Deviation from control (%) (b) |  |
| DMSO | 1 µM DOG | **** P< 0.0001 | **** P< 0.0001 | (a): Kruskal-Wallis test with Dunn’s post hoc test; (b): Mann-Whitney test |
| DMSO | 100 µM DOG | *** P= 0.0001 | **** P< 0.0001 |  |
| DMSO | 200 µM DOG | **** P< 0.0001 | **** P< 0.0001 |  |
| 1 µM DOG | 100 µM DOG | * P= 0.0461 |  | Kruskal-Wallis test with Dunn’s post hoc test |
| 1 µM DOG | 200 µM DOG | **** P< 0.0001 |  |  |
| 100 µM DOG | 200 µM DOG | **** P< 0.0001 |  |  |
| DMSO | 1 µM OAG | * P= 0.0387 | ** P= 0.0066 | (a): Kruskal-Wallis test with Dunn’s post hoc test; (b): Mann-Whitney test |
| DMSO | 100 µM OAG | **** P< 0.0001 | **** P< 0.0001 |  |
| DMSO | 200 µM OAG | **** P< 0.0001 | **** P< 0.0001 |  |
| 1 µM OAG | 100 µM OAG | **** P< 0.0001 |  | Kruskal-Wallis test with Dunn’s post hoc test |
| 1 µM OAG | 200 µM OAG | **** P< 0.0001 |  |  |
| 100 µM OAG | 200 µM OAG | **** P< 0.0001 |  |  |

**S. 6.** Mean values ± std. error of the latencies of the first bombykal-induced AP (in ms) (Fig. 7) during the first and last 20 min of recordings in the late activity phase and at rest in the presence or absence of different DAG-analogs.

| **Group** | **Late activity (ZT 1)** | | | **Rest (ZT 9)** | | |
| --- | --- | --- | --- | --- | --- | --- |
|  | n | beginning  (0-20 min) | end  (100-120 min) | n | beginning  (0-20 min) | end  (100-120 min) |
| DMSO | 6 | 16.73 ± 2.69 | 31.83 ± 6.59 | 7 | 17.58 ± 2.06 | 18.04 ± 2.23 |
| 100 µM DOG | 9 | 25.01 ± 2.59 | 56.17 ± 6.04 | 7 | 13.89 ± 1.17 | 64.21 ± 5.50 |
| 100 µM OAG | 6 | 13.33 ± 1.04 | 33.05 ± 5.30 | 7 | 12.96 ± 0.44 | 16.71 ± 1.27 |

**S. 7.** Mean values ± std. error of the late long-lasting pheromone response (given as number of APs in 295 s) (Fig. 8) during the first and last 20 min of recordings in the late activity phase and at rest in the presence or absence of different DAG-analogs.

| **Group** | **Late activity (ZT 1)** | | | **Rest (ZT 9)** | | |
| --- | --- | --- | --- | --- | --- | --- |
|  | n | beginning  (0-20 min) | end  (100-120 min) | n | beginning  (0-20 min) | end  (100-120 min) |
| DMSO | 6 | 625.2 ± 67.56 | 448.1 ± 35.50 | 7 | 764.1 ± 49.00 | 384.7 ±25.23 |
| 100 µM DOG | 9 | 366.3 ± 47.67 | 275.8 ± 23.30 | 7 | 521.2 ± 39.41 | 166.3 ± 23.31 |
| 100 µM OAG | 6 | 702.7 ± 77.77 | 352.7 ± 49.22 | 7 | 757.4 ± 38.53 | 548.9 ± 47.56 |

**S. 8.** Mean values ± std. error of the spontaneous activity without pheromone stimulation in the presence or absence of the DAG-analogs DOG and OAG (Fig. 9). Additionally the values of the percentage changes to the preceding control recordings are shown.

| **Group** | **Late activity (ZT 1)** | | | **Rest (ZT 9)** | | |  |
| --- | --- | --- | --- | --- | --- | --- | --- |
|  | n | absolute values (APs in 295 s) | Deviation from control (%) | n | absolute values (APs in 295 s) | Deviation from control (%) | |
| DMSO (before DOG) | 10 | 19.97 ± 6.04 |  | 10 | 72.65 ± 27.14 |  | |
| 100 µM DOG | 10 | 28.33 ± 11.48 | + 139.8 ± 104.9 | 10 | 51.64 ± 21.96 | -12.90 ± 14.97 | |
| DMSO (before OAG) | 7 | 40.00 ± 14.71 |  | 9 | 62.94 ± 20.04 |  | |
| 100 µM OAG | 7 | 73.00 ± 42.59 | + 148.0 ± 85.80 | 9 | 225.6 ± 114.4 | +289.7 ± 149.5 | |
